# LIN28 coordinately promotes nucleolar/ribosomal functions and represses the 2C-like transcriptional program in pluripotent stem cells

**DOI:** 10.1007/s13238-021-00864-5

**Published:** 2021-07-31

**Authors:** Zhen Sun, Hua Yu, Jing Zhao, Tianyu Tan, Hongru Pan, Yuqing Zhu, Lang Chen, Cheng Zhang, Li Zhang, Anhua Lei, Yuyan Xu, Xianju Bi, Xin Huang, Bo Gao, Longfei Wang, Cristina Correia, Ming Chen, Qiming Sun, Yu Feng, Li Shen, Hao Wu, Jianlong Wang, Xiaohua Shen, George Q. Daley, Hu Li, Jin Zhang

**Affiliations:** 1 Center for Stem Cell and Regenerative Medicine, Department of Basic Medical Sciences and the First Affiliated Hospital, Zhejiang University School of Medicine, Hangzhou, 310058 China; 2 Department of Molecular Pharmacology & Experimental Therapeutics, Center for Individualized Medicine, Mayo Clinic, Rochester, MN USA; 3 Tsinghua-Peking Center for Life Sciences, School of Medicine, Tsinghua University, Beijing, 100085 China; 4 The Black Family Stem Cell Institute and Department of Cell, Developmental and Regenerative Biology, Icahn School of Medicine at Mount Sinai, New York, NY 10029 USA; 5 Department of Biophysics and Department of Pathology of Sir Run Run Shaw Hospital, Zhejiang University School of Medicine, Hangzhou, 310058 China; 6 Department of Biological Chemistry and Molecular Pharmacology, Harvard Medical School, and Program in Cellular and Molecular Medicine, Boston Children’s Hospital, Boston, MA USA; 7 Stem Cell Transplantation Program, Division of Pediatric Hematology Oncology, Boston Children’s Hospital, Department of Biological Chemistry and Molecular Pharmacology, Harvard Medical School, Boston, MA USA; 8 College of Life Sciences, Zhejiang University, Hangzhou, 310058 China; 9 Department of Biochemistry, Zhejiang University School of Medicine, Hangzhou, 310058 China; 10 Institute of Life Science, Zhejiang University, Hangzhou, 310058 China; 11 Institute of Hematology, Zhejiang University, Hangzhou, 310058 China; 12 Zhejiang Laboratory for Systems and Precision Medicine, Zhejiang University Medical Center, Hangzhou, 310058 China

**Keywords:** LIN28, 2-cell-like program, nucleolar integrity, NCL/TRIM28 complex

## Abstract

LIN28 is an RNA binding protein with important roles in early embryo development, stem cell differentiation/reprogramming, tumorigenesis and metabolism. Previous studies have focused mainly on its role in the cytosol where it interacts with *Let-7* microRNA precursors or mRNAs, and few have addressed LIN28’s role within the nucleus. Here, we show that LIN28 displays dynamic temporal and spatial expression during murine embryo development. Maternal LIN28 expression drops upon exit from the 2-cell stage, and zygotic LIN28 protein is induced at the forming nucleolus during 4-cell to blastocyst stage development, to become dominantly expressed in the cytosol after implantation. In cultured pluripotent stem cells (PSCs), loss of LIN28 led to nucleolar stress and activation of a 2-cell/4-cell-like transcriptional program characterized by the expression of endogenous retrovirus genes. Mechanistically, LIN28 binds to small nucleolar RNAs and rRNA to maintain nucleolar integrity, and its loss leads to nucleolar phase separation defects, ribosomal stress and activation of P53 which in turn binds to and activates 2C transcription factor *Dux*. LIN28 also resides in a complex containing the nucleolar factor Nucleolin (NCL) and the transcriptional repressor TRIM28, and LIN28 loss leads to reduced occupancy of the NCL/TRIM28 complex on the *Dux* and rDNA loci, and thus de-repressed *Dux* and reduced rRNA expression. *Lin28* knockout cells with nucleolar stress are more likely to assume a slowly cycling, translationally inert and anabolically inactive state, which is a part of previously unappreciated 2C-like transcriptional program. These findings elucidate novel roles for nucleolar LIN28 in PSCs, and a new mechanism linking 2C program and nucleolar functions in PSCs and early embryo development.

**Supplementary Information:**

The online version contains supplementary material available at 10.1007/s13238-021-00864-5.

## INTRODUCTION

Embryonic stem (ES) cells are derived from the inner cell mass of the blastocyst of mouse early embryos and have the ability to differentiate into three germ layers and germ cells, but do not differentiate into trophectoderm and extraembryonic tissues, and therefore, are recognized as pluripotent. In contrast, embryonic blastomeres at the 2-cell stage have the potential to differentiate into all cell types of embryonic and extraembryonic tissues (Baker and Pera, 2018). The 2-cell state is characterized by activation of endogenous retrovirus or ERV genes which may act as alternative promoters or other regulatory elements for early developmental genes (Peaston et al., 2004). In cultured mouse ES cells, there is a rare and transient cell population with activated 2-cell marker genes including ERVs (Macfarlan et al., 2012). Understanding how ERVs are activated and repressed in the 2-cell state, and how these events influence zygotic genome activation and early embryo development are important questions that warrant further investigation. Recent work has shown that transcription or epigenetic factors can mediate ERV gene expression such as *Dux* (De Iaco et al., 2017; Hendrickson et al., 2017; Whiddon et al., 2017), *G9a* (Maksakova et al., 2013), *Suv39h* (Walter et al., 2016) and *Chaf1a* (Wang et al., 2018). However, other factors may contribute to turning-on or -off ERV genes. Furthermore, other features beyond epigenetics such as translational or metabolic status of these 2-cell like cells, and how these features impact the 2-cell like cell fate has yet been fully explored (Eckersley-Maslin et al., 2016).

After zygotic genome activation (ZGA) at the 2-cell stage, an embryo continues to develop through the four/eight-cell, morula and blastocyst stages, and prepares itself for implantation. As embryonic cells proliferate, basic anabolic metabolism and translational processes become more active. Nucleoli, the organelles involved in translation, functionally mature from nucleolar precursor bodies (NPB) during this process (Borsos and Torres-Padilla, 2016; Fulka and Aoki, 2016). Interestingly, it appears that shutting down ZGA and initiating nucleoli formation are not independent events, but interconnected, as exemplified by the recently reported TRIM28/NCL/LINE1 complex that can mediate both ZGA gene *Dux* repression and rRNA expression (Percharde et al., 2018). However, a complete picture on how the transition between ZGA and nucleolar formation occurs in the nucleus is not fully understood.

LIN28 is an RNA binding protein with important roles in early embryo development and in cultured ES cell differentiation and somatic cell reprogramming (Yu et al., 2007; Shinoda et al., 2013; Shyh-Chang and Daley, 2013; Zhang et al., 2016). In addition to its major molecular function in regulating *let-7* microRNAs, recent work has shown that it also binds to mRNAs to regulate translation (Cho et al., 2012; Wilbert et al., 2012), and may bind to DNA and influence transcription (Zeng et al., 2016). Most of the above studies have focused on LIN28’s cytoplasmic role, with fewer studies focusing on LIN28 nuclear functions (Piskounova et al., 2011; Zeng et al., 2016). Even though LIN28 has been described to localize in the nucleolus under certain contexts, no functional roles have been ascribed to LIN28 in this sub-nuclear compartment (Kim et al., 2014). Moreover, the role of LIN28 in the pre-implantation embryo remains unclear. Here, we show that *Lin28a* mRNA and protein have a dynamic expression pattern in pre- and post-implantation embryo development. It regulates both the 2C/ERV gene expression and rRNA expression through a nucleolus-related mechanism. Our findings for the first time elucidate a novel role of nucleolar LIN28 in mediating the 2C-like and ES state homeostasis in cultured cells, and suggest that LIN28 is a new molecular switch from ERV-expressing 2C/4C stage to nucleolus-matured morula/blastocyst stage during murine pre-implantation embryo development.

## RESULTS

### Temporal and spatial expression of LIN28A in mouse early embryo development and pluripotent stem cells

**Figure 1:**
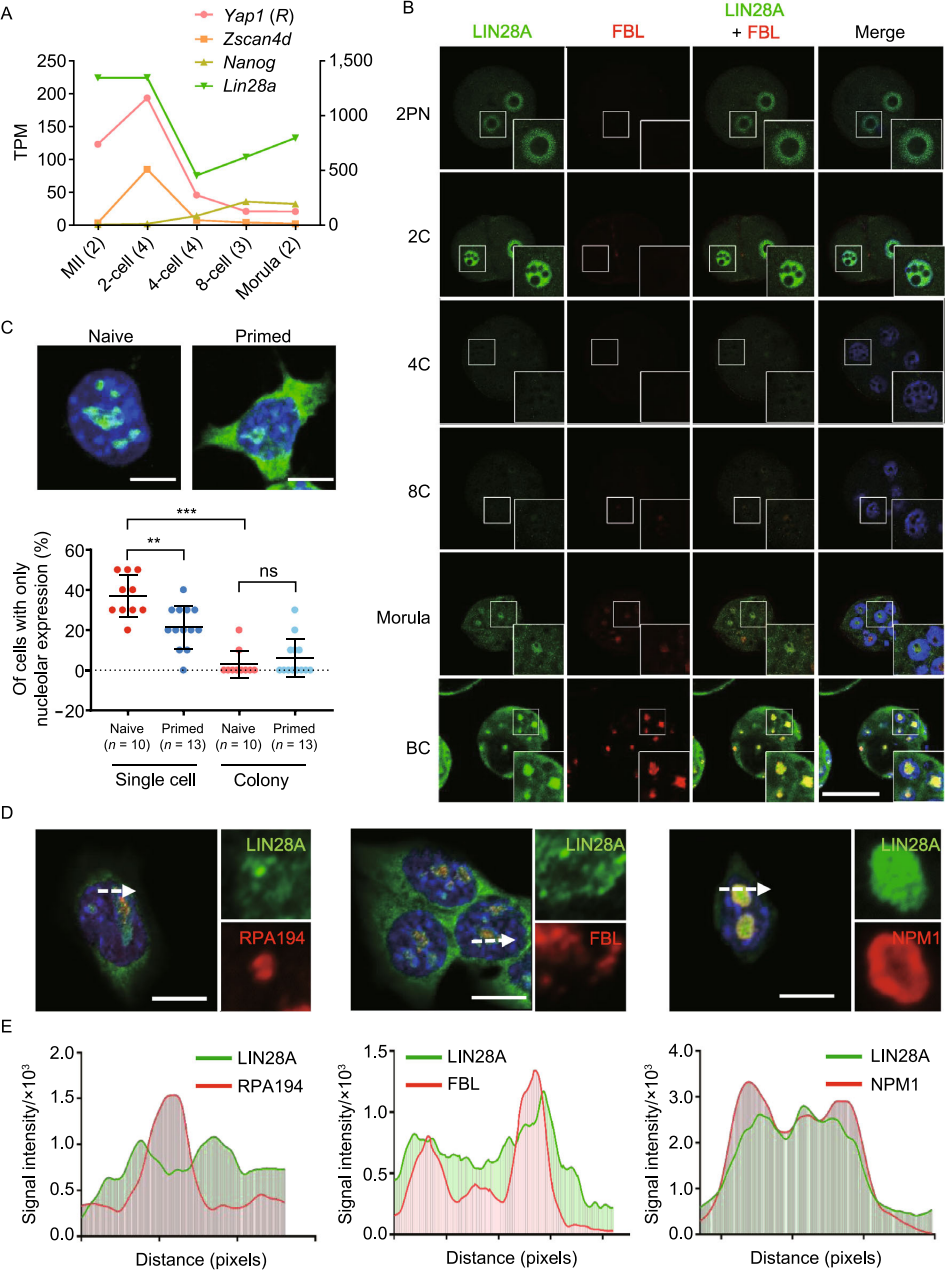
**Temporal and spatial expression of LIN28A in mouse early embryo development and different states of pluripotent stem cells**. (A) mRNA expression of *Lin28a* and stage-specific marker genes *Yap1* (oocyte), *Zscan4d* (2C) and *Nanog* (inner cell mass of blastocyst) at different developmental stages analyzed from published RNA-seq data (Wang, et. al., 2018). MII: metaphase II. TPM: transcripts per million for mRNA expression level. R: right y-axis. Numbers in the parentheses mean the number of embryos. (B) Immunofluorescence staining of LIN28A and nucleolar marker protein Fibrillarin (FBL) in the early stages of mouse pre-implantation embryo development. 2PN: two pronuclei stage. BC: blastocyst stage. Scale bar, 50 μm. (C) Upper panels: Representative Lin28A immunostaining in naïve and primed pluripotent stem cells. Lower panel: summary of the percentage of cells that have LIN28A mainly in the nucleolus. Cells were grown as single cells, or in small colonies and were counted separately. Each dot in the plot represents one counting experiment of around 10 cells in each condition. Scale bar, 10 μm. **P < 0.01, ***P < 0.001, *t*-test, error bar: standard error of the mean. (D and E) Immunofluorescence staining and confocal (with Airyscan module) showing LIN28A sub-cellular localization and nucleolar compartment marker proteins such as Nucleophosmin (NPM1, granular component), Fibrillarin (FBL, dense fibrillar component) and Pol I (RPA194, fibrillar center). Histograms represent signal intensity along the arrow bars for each protein. Scale bar, 10 μm

We first inspected LIN28A expression at both mRNA and protein levels in pre-implantation embryos. *Lin28a* mRNA level is high at oocyte and pronuclear zygote stages, but decreased sharply after the 2-cell stage (Fig. 1A). Afterwards, its expression increased steadily as embryos progress through 4-cell, 8-cell, morula stages, and reach blastocysts, which is consistent with LIN28A expression in the inner cell mass-derived embryonic stem cells (Fig. 1A). In contrast, the ZGA marker gene *Zscan4d* is only expressed at the 2-cell stage, and gets completely silenced after the 4-cell stage (Fig. 1A). LIN28A immunostaining analysis in embryos derived at different stages *in vivo* confirmed the RNA-seq results (Figs. 1B and S1A). LIN28A protein is expressed and mainly localized in the immature nucleolus precursor bodies before the 2-cell stage, is lower at late 2-cell and 4-cell stages, and is re-expressed along the eight-cell and morula stages in the forming nucleolar region. Upon entering the blastocyst stage LIN28A becomes abundant in both nucleoli and cytoplasm (Figs. 1B and S1A). Next, we inspected the endogenous retrovirus MERVL-Gag protein which was expressed predominantly at the 2-cell/4-cell stage and broadly reduced thereafter (Fig. S1B). This reciprocal expression pattern of ERV compared to LIN28A protein suggests that LIN28A may play a role in repressing ERV expression.

Furthermore, we examined LIN28A expression in post-implantation embryos. Besides high-level expression at mRNA level (Fig. S1C), we found that LIN28A is mainly cytoplasmic in post-implantation embryos, which greatly contrasts with early pre-implantation stage embryos (Fig. S1D).

Because naïve and primed cells *in vitro* correspond with pre- and post-implantation epiblast cells, we determined LIN28A localization in these two types of pluripotent stem cells in culture. We observed that when cells are in colonies, in either naïve or primed state, LIN28A localizes both in the cytoplasm and the nucleolus; however, in isolated single cells, LIN28A protein is more likely to be localized in the nucleolus in naïve state cells compared with primed state cells (Figs. 1C and S1E). We further examined LIN28A nucleolar compartmentalization by immunostaining. It appears that LIN28A overlaps with the granular component (GC, labeled by NPM1) where ribosome assembly takes place, and with the inner dense fibrillar component (DFC, marked by Fibrillian or FBL) where rRNA is modified, and has the least overlap with the core of fibrillar center (FC, marked by RNA Pol I) where rRNA is transcribed (Figs. 1D, 1E and S1F). This unique pattern of LIN28A nucleolar localization, and the associated dynamic nucleoli-cytoplasm changes, strongly suggest that LIN28 may have novel and versatile roles in these compartments at multiple key stages during early embryo development.

### Genes activated at 2-cell stage are induced in *Lin28* knockout pluripotent stem cells

**Figure 2:**
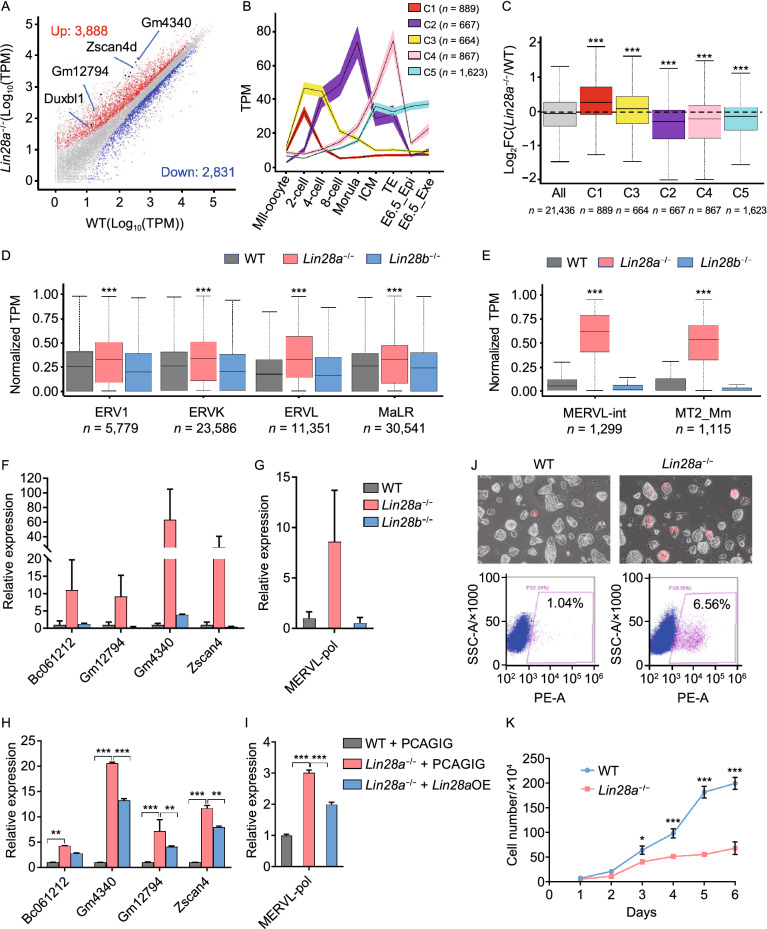
**Deficiency of LIN28 causes activation of ERV and 2C marker genes**. (A) Scatter plot of RNA-sequencing data comparing gene expression of wild-type and *Lin28a* knockout ES cells. (B) Embryo stage specific genes were classified into five clusters based on their expression pattern. (C) Log_2_(fold change) of gene expression abundance between *Lin28a* knockout ES cells and wild-type cells for each gene cluster. ***P < 0.001, Mann-Whitney U test. (D) Different classes of Long Terminal Repeat or LTR expression in *Lin28a* and *Lin28b* knockout ES cells. (E) Two sub-classes of ERVL genes MERVL-int and MT2_Mm are significantly up-regulated in *Lin28a* knockout ES cells. For (D and E), the TPM values of LTR expression were linearly normalized to a range from 0 to 1 across all samples. ***P < 0.001, Wilcox signed rank test. (F and G) qPCR validation of selected 2C marker genes and Mervl-polymerase gene (*Mervl-pol*) in wild-type and *Lin28a/b* knockout ES cells. (H and I) LIN28A overexpression in knockout cells reversed the activation of 2C genes and ERV genes. (J) Microscopy pictures and flow cytometry showing the percentage of 2C-like cells using ES cells transduced with a *2C::tdTomato* reporter construct. (K) Proliferation curve of wild-type and *Lin28a* knockout ES cells. *P < 0.05, **P < 0.01, ***P < 0.001, two-way ANOVA, *n* = 3, error bar: standard error of the mean

In order to examine how LIN28 can influence gene expression, we performed RNA-seq analysis using three different settings of wild-type and *Lin28*-deficient pluripotent stem cells: 1) knockout ES single clones derived with CRISPR/CAS9, 2) ES cells derived from *Lin28* knockout mice, and 3) iPS cells derived from *Lin28* knockout MEF cells (Fig. S2A). We found that *Lin28a* knockout induced up-regulation of 2,418 genes (log_2_-fold change > 1, *P*-value < 0.05) in ES cells cultured in the LIF/2i naïve condition, including some 2-cell/4-cell stage marker genes *Zscan4d*, *Duxbl1*, *Zfp352*, *Gm4340*, *Gm12794* (Fig. 2A and Table S1). Using published RNA-seq data (Wang et al., 2018), we clustered highly expressed genes across each stage of mouse pre-implantation embryo development (Fig. 2B), and found that knockout of *Lin28a* increased 2-cell expressing cluster 1 (C1) and 2-cell/4-cell expressing cluster 3 (C3) genes, and decreased genes expressed in later stages (C2, C4, C5 clusters) (Fig. 2C). The 2-cell/4-cell stage ZGA is characterized by activation of transposable elements (TEs), particularly ERVL subclasses MERVL-int and MT2_Mm (Fig. S2B–E). We systematically examined ERV genes and found global up-regulation of each LTR class (Fig. 2D), particularly MERVL-int and MT2_Mm sub-classes in *Lin28a* knockout ES cell lines (Figs. 2E and S2F). The up-regulated expression of 2C genes including *Zscan4d*, *Gm4340*, *Gm12794*, *Bc061212*, and MERVL were further validated by qRT-PCR (Fig. 2F and 2G). Furthermore, these effects were reversed by transiently over-expressing *Lin28a* in each cell line (Figs. 2H, 2I and S2G). Interestingly, *Lin28b* knockout did not show the same up-regulation (Figs. 2F and 2G), likely because ES cells predominantly express *Lin28a* (Zhang et al., 2016). For *Lin28* knockout MEF cell reprogramming, we found in *Lin28a*/*b* double knockout cells, the reprogrammed iPS cells resembled ES cells in terms of their activated ZGA gene sets (Fig. S2H) and ERV expression (Figs. S2I–K), and this effect was rescued by overexpressing *Lin28a* (Fig. S2L and S2M), demonstrating induced pluripotency needs LIN28 to repress ERV expression (Friedli et al., 2014). Considering that the cells used in the experiment were cultured in LIF/2i condition, we also compared the expression of 2C genes and ERV genes in *Lin28* knockout or WT PSCs transitioned from LIF/2i to LIF/serum condition, and found the same 2C and ERV gene upregulation in *Lin28* knockout ES (Fig. S2N) and iPS cells (Fig. S2O). To further verify these findings, we used CRISPR/CAS9 to generate *Lin28a* knockout single clones in another ES cell line E14 (Fig. S3A and S3B), and also examined *let-7* overexpression ES cells in which *Lin28a* level is repressed (Fig. S3C). We observed a similar trend of up-regulation of 2C/4C marker genes and MERVL-int and MT2_Mm ERVs (Fig. S3D–F). Together, these results demonstrate that LIN28 deficiency induces a 2C/4C-like state transcriptional program in PSCs.

It was previously reported that in cultured ES cells a small population of 2C-like cells show fluctuating expression of ERV genes (Macfarlan et al., 2012). Thus, we wonder if in *Lin28* knockout cell culture, more cells are induced to 2C-like cells, or alternatively if the same number of existing 2C-like cells show an increase of 2C gene expression. Using a *2C::tdTomato* reporter in which a *tdTomato* gene is under control of a MERVL promoter (Macfarlan et al., 2012), we examined 2C status of individual cells by flow cytometry. *Lin28* knockout ES or iPS cells showed both increased percentage of *tdTomato* positive cells, and increased signal intensity (Figs. 2J and S3G), suggesting that both a population shift toward the 2C-like state and enhancement of ERV expression took place. Lastly, and consistent with previous reports, *Lin28* knockout induced a decrease of cell proliferation in both ES and iPS cell lines (Figs. 2K and S3H) (Zhang et al., 2016), and an altered cell cycle, with an increase in G_2_/M phase (Fig. S3I) (Xu et al., 2009). Together, these results demonstrate that LIN28 represses ERV expression and the 2C transcriptional program in PSCs.

### LIN28 regulates ERV expression through repression of *Dux*

**Figure 3:**
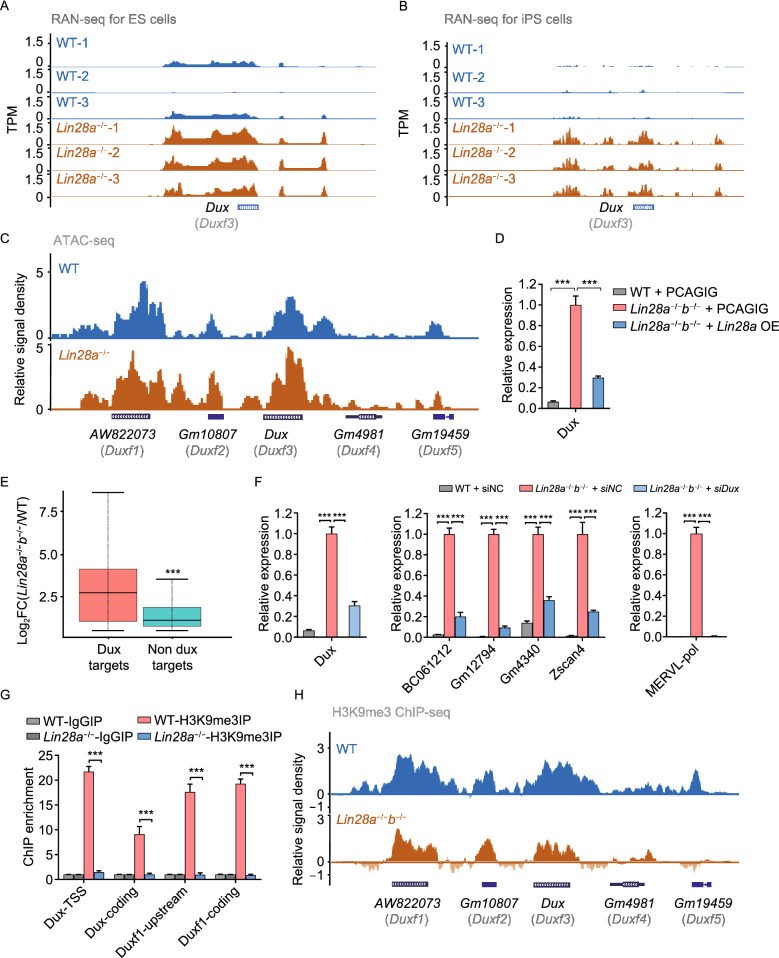
**LIN28 regulates ERV expression through repression of** ***Dux***. (A and B) UCSC Genome Browser view showing RNA-seq results at Dux gene in the indicated ES cells (A) or iPS cells (B). (C) ATAC-seq tracks at the *Dux* family loci in wide-type (blue) and Lin28a knockout (brown) ES cells. (D) qRT-PCR showing *Dux* gene expression in wild-type and double knockout iPS cells transduced with empty vector or Lin28a overexpressing vector (relative expression normalized to Gapdh). (E) Boxplot showing that Dux targets are significantly more induced than non-targets by analyzing the upregulated genes in Lin28 double knockout iPS cells. (F) qRT-PCR showing *Dux*, 2C gene and MERVL expression in wild-type and Lin28 double knockout iPS cells treated with scramble negative control or the *Dux* siRNAs. (G) ChIP-qPCR showing H3K9me3 levels for *Dux* gene locus in wild-type and knockout ES cells. IgG was used as control. (H) UCSC Genome Browser view of H3K9me3 ChIP-seq results for the *Dux* family. *P < 0.05, **P < 0.01, ***P < 0.001, two-way ANOVA, *n* = 3, error bar: standard error of the mean

We next investigated how LIN28 regulates the 2C program in pluripotent stem cells. Recent studies have described that a pioneer transcription factor, the DUX protein, directly binds to promoters and LTR elements on 2C genes and repetitive elements, and activates their transcription in cultured PSCs (De Iaco et al., 2017; Hendrickson et al., 2017; Whiddon et al., 2017), and *Dux* gene knockout in mice has minor effects on ZGA in developing embryos (Chen and Zhang, 2019; Guo et al., 2019). RNA-seq and ATAC-seq data support that *Dux* expression was markedly up-regulated in Lin28 knockout cells (Fig. 3A–C), and the expression levels can also be rescued by overexpressing LIN28A (Fig. 3D). Furthermore, we found that *Dux* target genes are among the most highly activated genes upon *Lin28* knockout (Fig. 3E), and *Dux* depletion significantly abrogated their activation (Fig. 3F). As one of the mechanistic role of DUX is to open up chromatin (Hendrickson et al., 2017), we examined ATAC-seq results with wild-type and *Lin28* knockout cells, and found increased chromatin accessibility at loci of those up-regulated genes, particularly at MERVL-int and MT2_Mm (Fig. S4A). Moreover, we examined H3K9 trimethylation which is a marker for repressed *Dux* genes, and found *Dux* and *Duxf1* showed a reduced level of H3K9me3 in *Lin28a* knockout ES cells (Fig. 3G) and *Lin28a*/*b* double knockout iPS cells (Fig. 3H). Also, the H3K9me3 levels were decreased at the *Dux* downstream targets (Hendrickson et al., 2017; Whiddon et al., 2017) determined by ChIP-seq (Fig. S4B and S4C) or ChIP-qPCR (Fig. S4D) in both ES and iPS cells. Given that LIN28 interacts with TET1, we also examined DNA methylation or hydroxymethylation level with publicly available data (Zeng et al., 2016), and did not find significant difference at the *Dux* loci in *Lin28* knockout ES cells and wild-type cells (Fig. S4E). Together, these results demonstrate that *Lin28*-deficiency induced a 2C-like transcriptional and epigenetic program in cultured PS cells mainly through upregulation of *Dux*.

### LIN28 binds to RNAs in the nucleolus, and its loss leads to nucleolar/ribosomal abnormalities

**Figure 4:**
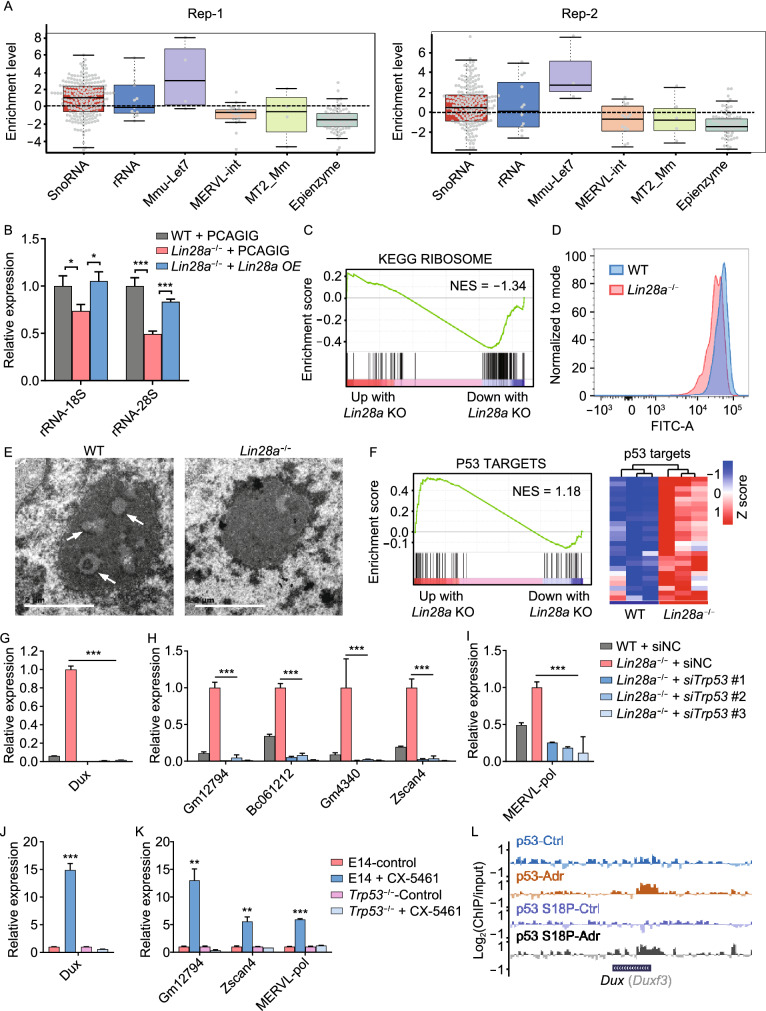
**LIN28 binds to snoRNA and rRNA, and its loss leads to reduced rRNA biogenesis and ribosomal stress**. (A) LIN28A CLIP-seq enrichment analysis. ERVL, MERVL-int and MT2_Mm classes were not enriched in LIN28A binding targets compared with other genes. (B) qRT-PCR showing the expression of rRNA in wild-type and *Lin28a* knockout ES cells. PCAGIG: empty vector. OE: overexpression. *P < 0.05, ** *P* < 0.01, *** *P* < 0.001, two-way ANOVA, *n* = 3, error bar: standard error of the mean. (C) GSEA analysis showing collective changes in the Ribosome gene set in *Lin28a* knockout ES cells compared with wild-type cells. NES: normalized enrichment score. (D) Flow cytometry showing OP-puromycin (OP-puro) labeling of wild-type and *Lin28a* knockout ES cells. (E) Electro-microscopy showing nuclear morphology of wild-type and *Lin28a* knockout ES cells. Scale bar, 2 μm. (F) Left panel: GSEA analysis showing collective changes in the p53 signaling pathway gene set. Right panel: up-regulated p53 target genes in *Lin28a* knockout ES cells compared with wild-type cells. (G–I) qRT-PCR showing *Dux* (G), 2C-related genes (H) or MERVL (I) expression in wild-type and *Lin28a* knockout ES cells treated with scramble negative control, or *Trp53* siRNAs. (J and K) qRT-PCR showing the expression of *Dux* (J) or 2C-related genes (K) in ES cells treated with 4 μmol/L CX-5461 for 12 h or equal water (control), or in *Trp53* knockout ES cells treated with the same conditions. (L) UCSC Genome Browser view of ChIP-seq results using antibodies of p53 and phosphorylated p53 S18P for *Dux* gene locus. *P < 0.05, **P < 0.01, ***P < 0.001, two-way ANOVA, *n* = 3, error bar: standard error of the mean

Next, we further explored the mechanistic role of LIN28 in regulating ERV and *Dux* repression. Given that LIN28 is an RNA binding protein, we performed CLIP-seq experiments using an antibody against LIN28A with wild-type mouse ES cells. However, we did not observe a significant enrichment of direct LIN28A binding to the RNAs of ERVs or epigenetic enzymes compared with *let-7* microRNA precursors (Fig. 4A). Surprisingly, LIN28A binding targets showed a strong enrichment in small nucleolar RNA (snoRNA) and rRNA (Figs. 4A and S5A). Similar enrichment in snoRNA and rRNA binding was validated using a published CLIP-seq dataset in A3-1 mES cell line (Fig. S5B and S5C) (Cho et al., 2012). LIN28A appears to pull down snoRNAs, rather than their host genes whose introns harbor snoRNAs (Fig. S5D). RNA immunoprecipitation (RIP) experiments using nuclear and cytoplasmic fractions showed that the majority of interactions took place in the nuclear fraction but not the cytoplasm (Fig. S5E). In contrast, a previously identified LIN28A cytoplasmic target *Ndufd8* (Zhang et al., 2016) did not show enrichment in immunoprecipitated nuclear fraction (Fig. S5E and S5F). Interestingly, even though the total LIN28A expression level was lower in the naïve state ES cells compared to the primed state (Zhang et al., 2016), its interaction with snoRNAs and rRNA was higher (Fig. S5E and S5F), consistent with a preferential localization of LIN28A protein in the nucleolus in the naïve state cells (Fig. 1C).

LIN28 interacts with microRNA *let-7* and mediates its processing and metabolism (Chang et al., 2013; Piskounova et al., 2011), and given that LIN28 also interacts with rRNA and snoRNA, and snoRNAs are engaged in processing rRNA (Kiss, 2002), we examined mature rRNAs including 18S and 28S, and found that knockout cells had significantly reduced levels (Figs. 4B and S5G), and the defects can be rescued by overexpressing LIN28A (Fig. 4B). Gene set enrichment analysis (GSEA) showed that *Lin28* knockout also reduced ribosomal biogenesis (Figs. 4C and S5H) and translation activity (Figs. 4D and S5I) in both ES and iPS cells. Indeed, LIN28’s interaction with snoRNA and rRNA is consistent with its localization in the nucleolar DFC region where snoRNA-mediated rRNA modification takes place (Fig. 1D). Using electron microscopy, we observed that *Lin28* knockout cells displayed reduced electron density at the DFC “ring” structure (Figs. 4E and S5J), suggesting functional abnormalities at the DFC in the nucleolus in knockout cells. As the nucleolus and its sub-compartments represent liquid phase assembled by RNA and RNA binding ribonucleoproteins (Feric et al., 2016), we used fluorescence recovery after photobleaching (FRAP) assays to analyze wild-type and *Lin28* knockout ES cells transduced with FBL-mCherry, and we found reduced recovery of FBL after photobleaching in the knockout cells (Fig. S5K), indicating disrupted phase separation of nucleolar DFC. Together these data demonstrate LIN28 interacts with RNAs in the nucleolus, and its loss leads to nucleolar abnormalities including defects in nucleolar phase separation.

### LIN28 deficiency promotes *Dux*/2C program by nucleolar/ribosomal stress-induced p53 activation

It is well-established that nucleolar abnormalities or nucleolar/ribosomal stress is associated with p53 activation (Lohrum et al., 2003; Dai and Lu, 2004; Dai et al., 2004; Golomb et al., 2014; Yang et al., 2018), and we thus asked whether p53 signaling was activated in the *Lin28* knockout PSCs. As expected, we found that multiple direct p53 targets are elevated in knockout ES cells (Fig. 4F) and iPS cells (Fig. S5L). Surprisingly, when we used three different siRNAs to knockdown *Trp53* in *Lin28* knockout cells, it reversed the upregulation of *Dux* and 2C genes, including MERVL (Fig. 4G–I). These results together suggest that *Dux* and 2C gene activation in LIN28-defieicnt cells may be caused by nucleolar/ribosomal stress and p53 activation. To further test this hypothesis, we directly treated wild-type ES cell with RNA Pol I inhibitor CX-5461, which decreased rRNA levels and induced ribosomal stress (Fig. S5M), similar to *Lin28* knockout. We found that *Dux* and 2C gene and MERVL expression were strongly induced as well as p53 protein and its targets (Figs. S5N-P), recapitulating the *Lin28* knockout phenotype. When the stress signaling was blocked by knocking out *trp53*, the activated *Dux* and 2C genes and MERVL were rescued (Figs. 4J, 4K and S5Q). To explore whether p53 directly regulates *Dux* or 2C gene expression, we analyzed p53 ChIP-seq data (Li et al., 2012), and found that p53 bound to the *Dux* locus in ES cells treated with adriamycin, a DNA damage agent widely used to activate p53, but not other 2C gene such as *Zscan4d* (Figs. 4L and S5R). Taken together, these data demonstrate that LIN28 regulates nucleolar integrity, and in the *Lin28* knockout cells, nucleolar stress-activated p53 and its binding and regulation on *Dux* contribute to the 2C program.

### LIN28 maintains nucleolar integrity through physical association with nucleolar/ribosomal proteins

**Figure 5:**
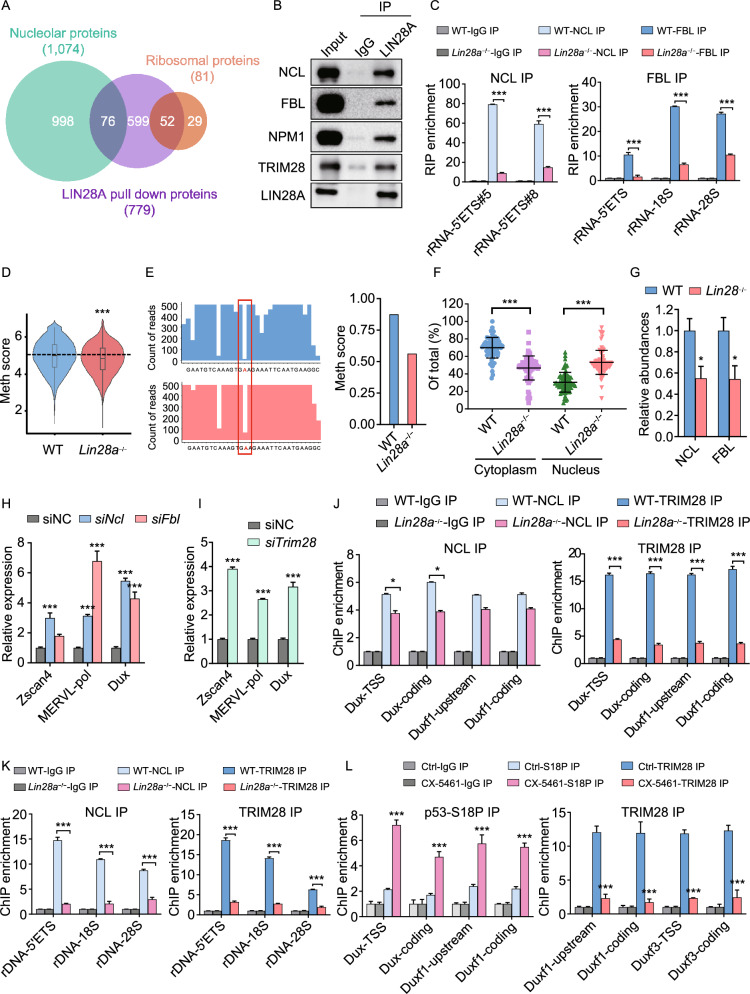
**LIN28A is associated with nucleolar proteins and mediates rRNA biogenesis and 2C gene repression through the NCL/TRIM28 complex**. (A) Venn diagrams showing the overlap between proteins pulled down by LIN28A and nucleolar proteins or ribosomal proteins. (B) Validation of the interaction between the endogenous LIN28A and nucleolar or nuclear proteins by Co-IP followed by Western blot analysis in ES cells. IgG was used as a negative control for the IP. (C) RIP-qPCR assays for NCL and FBL in wild-type and *Lin28a* knockout ES cells. *P < 0.05, **P < 0.01, ***P < 0.001, two-way ANOVA, *n* = 3, error bar: standard error of the mean. (D) Quantification of 2’-O-Me by RiboMethseq for all bases in 28s, 18s and 5s rRNAs in wild-type and *Lin28a* knockout ES cells. MethScore was calculated using the method mentioned in (Birkedal et al., 2015; Marchand et al., 2016). ***P < 0.001. Wilcox signed rank test. (E) Left panel: RiboMeth-seq showing profiles of mapped sequencing reads in a range of a snoRNA targeting region on the 28s rRNA (from 3,366 bp to 3,397 bp). Red highlighted A is a predicted methylation site (from SnoRNA Orthological Gene Database). Right panel, calculated RiboMeth-Seq score to indicate the level of the 2’-O-methylation at the predicted site of A (Birkedal et al., 2015). (F) Proteomics analysis showing the nucleus and cytoplasm proportion of ribosomal proteins in wild-type and *Lin28a* knockout ES cells. ***P < 0.001, *t*-test, *n* = 3, error bar: standard error of the mean. (G) Proteomics analysis showing the relative abundances of indicated proteins in wild-type and *Lin28* knockout cells (averaged proteomics data from *Lin28a* knockout ES cells and *Lin28a*/*b* knockout iPS cells). Error bar: standard error of the mean. *n* = 2 independent proteomics experiments. (H and I) qRT-PCR showing the expression of 2C-related genes in ES cells treated with scramble negative control or the indicated siRNAs. (J and K) NCL and TRIM28 ChIP-qPCR analysis at the *Dux* (J) and rRNA (K) loci in wild-type and *Lin28a* knockout ES cells. (L) p53 and TRIM28 ChIP-qPCR analysis at the *Dux* loci in untreated and CX-5461-treated ES cells

In order to further understand how LIN28 maintains nucleolar and ribosomal integrity, we investigated the protein components of the LIN28-containing complex in the nucleus, by performing LIN28A immunoprecipitation followed by mass spectrometry with cytosolic and nuclear fractions of wild-type ES cells (Fig. S6A). LIN28A nuclear interacting partners were mostly enriched with proteins in ribosome biogenesis, rRNA processing and ribonucleoprotein complex biogenesis, etc. (Fig. S6B). Indeed, almost 10% LIN28A interacting proteins were nucleolar proteins, and a total of 64% ribosomal proteins (RPs) were pulled down by LIN28A (Fig. 5A), including nucleolar components such as NCL, FBL and NPM1 (Figs. 5B and S6C), as well as ribosomal proteins such as RPL5, RPL23, RPL26, RPS6, RPS7, RPS19 (Fig. S6D). Interestingly, we also found the interaction between LIN28A and nucleolar proteins such as NCL is RNA-dependent (Fig. S6E), which is reminiscent of the RNA-mediated phase separation of nucleolar sub-compartments (Feric et al., 2016). As NCL, an rRNA binding protein, plays a role in the rRNA synthesis and processing (Ginisty et al., 1998; Mongelard and Bouvet, 2007), we performed RNA immunoprecipitation (RIP) and qPCR, and found that the level of NCL protein bound to pre-rRNA was significantly reduced upon *Lin28* knockout in ES cells (Fig. 5C). Moreover, FBL is the rRNA methyl-transferase in the DFC, and it binds to the C/D Box RNPs and catalyzes 2’-O-methylation on the target rRNA (Tollervey et al., 1993). *Lin28* knockout also reduced the occupancy of FBL protein on rRNA (Fig. 5C). Correspondingly, the level of 2’-O-methylation was also decreased in *Lin28a* knockout ES cells, compared with wild-type cells (Fig. 5D and 5E), which is also consistent with the rRNA reduction (Figs. 4B and S5G). Together, these data demonstrate that LIN28A coordinates with NCL, FBL and nucleolar RNAs to promote rRNA biogenesis, which all contributes to the nucleolar integrity. Moreover, proteomics analysis showing *Lin28a* knockout led to mis-localization of ribosomal proteins from cytosol to nucleus (Figs. 5F, S6F and S6G), likely because the disrupted nucleolar/ribosomal integrity impeded ribosome assembly and transport to the cytosol, and mis-localized ribosomal proteins in the nucleoplasm can sequester MDM2, and cause p53 activation (Lohrum et al., 2003; Dai and Lu, 2004; Zhang and Lu, 2009; Golomb et al., 2014). We thus performed a cycloheximide-chase analysis of protein degradation and found *Lin28a* knockout cells had prolonged half-life of the p53 protein (Fig. S6H). Together, these data demonstrate that the interactions between LIN28 and nucleolar proteins or ribosomal proteins maintain nucleolar/ribosomal integrity and prevent cells from nucleolar stress and p53 activation.

### Loss of LIN28A releases *Dux* repression by the NCL/TRIM28 complex

We next examined the abundance of the key nucleolar proteins that also interact with LIN28A, and found reduced total protein level of NCL, FBL upon LIN28A depletion (Fig. 5G). NCL is mostly enriched in the DFC and GC region of the nucleolus (Biggiogera et al., 1990; Mongelard and Bouvet, 2007; Jia et al., 2017), and immunofluorescence revealed that *Lin28* knockout led to disappearance of the NCL-marked “ring” structure in ES cells (Fig. S6I), reminiscent of the electron microscopy results showing reduced “ring” structure above (Figs. 4F and S5J), suggesting LIN28A has a critical role in maintaining the nucleolar DFC and GC regions. NCL forms a complex with TRIM28, and resides within a peri-nucleolar chromatin region containing *Dux* locus to mediate both *Dux* repression and rRNA expression (Percharde et al., 2018). We thus explored whether LIN28 can also regulate *Dux* and rRNA through this mechanism. To our surprise, LIN28A also pulled down TRIM28 (also known as KAP1) (Figs. 5B and S6C), which directly binds and represses *Dux* (Percharde et al., 2018), suggesting LIN28A is in the same repressive complex containing TRIM28 and NCL, although its depletion does not affect the level of TRIM28 protein (Fig. S6J). To further investigate whether the effects of *Lin28* knockout is through the NCL/TRIM28 complex, we first knocked down NCL, FBL and TRIM28 with multiple siRNAs for each gene (Fig. S6K), and they all recapitulated the activated 2C transcriptional program indicated by MERVL and *Dux* expression and rRNA repression as well as p53 activation (Figs. 5H, 5I, S6L and S6M). Most importantly, *Lin28* knockout led to reduced occupancy of TRIM28 and NCL proteins on the *Dux* locus (Fig. 5J) and the rDNA locus (Fig. 5K), suggesting that the *Dux* and rRNA regulation by NCL/TRIM28 complex is LIN28-dependent. Besides, Pol I inhibitor CX-5461 treatment also recapitulated *Lin28* knockout ES cells in the phenotypes of reduced TRIM28 occupancy and increased p53 occupancy on the *Dux* locus (Fig. 5L), demonstrating a conserved *Dux*/2C activation mechanism through either genetically or pharmacologically disrupting nucleolus.

Together, these data demonstrate that LIN28, the NCL/TRIM28 complex and the *Dux* loci appears to reside within the same peri-nucleolar compartment, and the regulation of *Dux* gene suppression and rRNA expression conferred by NCL/TRIM28 is dependent on LIN28. Considering the nucleolar stress and p53 mechanism revealed before, our data together elucidated two parallel pathways downstream of nucleolar stress, namely, p53-activated *Dux* expression and NCL/TRIM28 de-repressed *Dux* expression, both upon nucleolar disruptions in *Lin28* knockout cells (Fig. S6N).

### LIN28 maintains population homeostasis of low RP gene-expressing 2C-like cells in ES cell culture

**Figure 6:**
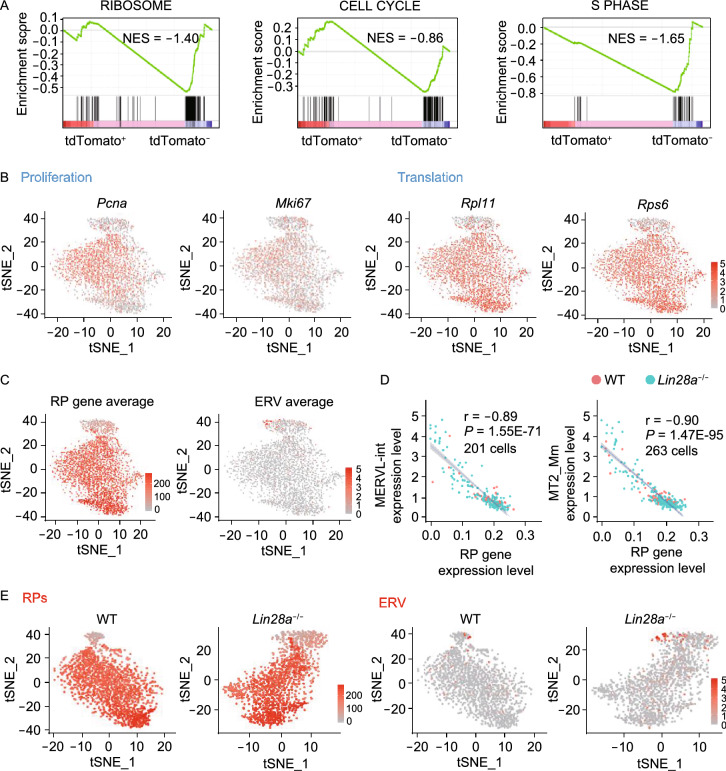
**Loss of LIN28 alters 2-cell/ES homeostasis, and gives rise to more low RP expressing and translationally inert 2C-like cells**. (A) GSEA analysis of *2C::tdTomato+* ES cells compared with *2C::tdTomato-* cells for the indicated gene sets. (B) tSNE plots showing indicated gene expression with wild-type and *Lin28a* knockout ES cells put together. (C) tSNE plot showing averaged ribosomal protein (RP) gene expression and ERV gene expression with the same cells as in (B). The color intensity represents the averaged expression of ERV genes or ribosomal protein genes in a single cell. (D) Scatter plots showing the correlation between MERVL-int or MT2_Mm expression and ribosomal gene expression by single cell RNA-seq in wild-type and *Lin28a* knockout cells. Each dot represents a single cell with detectable ERV expression. (E) tSNE plots showing the expression of ribosomal protein genes or ERV genes by single cell RNA-seq in wild-type and *Lin28a* knockout ES cells, respectively. The color intensity represents the averaged expression of ERV genes or ribosomal protein genes in a single cell

To validate whether the above nucleolus and ribosome-related mechanisms can explain the dynamic population homeostasis of ES and 2C-like cell cultures in the wild-type condition, we first sorted the *2C::tdTomato* positive 2C-like cell population and analyzed its transcriptome, and found significantly reduced ribosomal protein gene and cell cycle gene categories compared with *2C::tdTomato* negative cells (Fig. 6A), suggesting these 2C-like cells are slowly cycling and translationally inert cells. To understand the homeostasis at a single cell level, we performed single cell RNA-seq with wild-type and *Lin28a* knockout ES cells (Fig. S7A and S7B), and found that the population with higher MERVL-int and MT2_Mm expression tends to not express proliferation marker genes *Pcna* and *Mki67*, nor ribosomal protein genes such as *Rpl11* and *Rps6* (Fig. 6B). Indeed, the ERV gene expression showed strong negative correlation with ribosomal protein genes (Figs. 6C, 6D, S7C and S7D). Strikingly, *Lin28a* knockout cells led to an expansion of the low RP gene-expressing, slowly cycling and translationally inert 2C-like population (Fig. 6E). Lastly, when we dissected the cell cycle status of ERV-expressing cells, we found that the knockout-induced 2C-like cells are mostly enriched in the G_1_ and G_2_/M phases, but not in S phase (Fig. S7E), consistent with the fact that loss of LIN28A give rise to more of the slowly proliferating cells. Together, these results validated that at cell population level, LIN28’s regulation on 2C-like/ES cell homeostasis is associated with the nucleolar and ribosomal mechanisms elucidated above.

### LIN28 facilitates 2C program repression and promotes ribosomal genes during the 2-cell/4-cell to morula/blastocyst stage transition of mouse pre-implantation embryos

**Figure 7:**
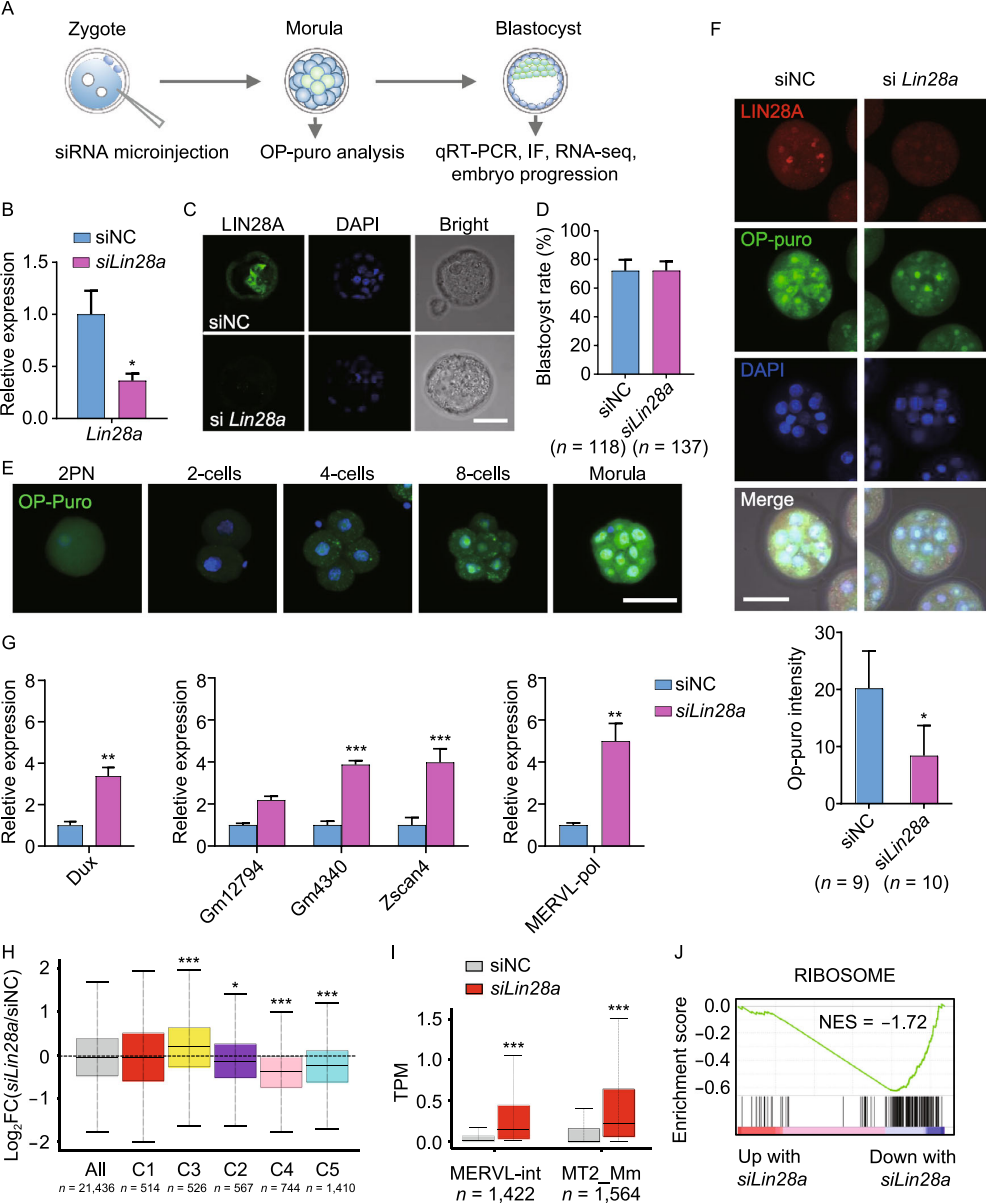
**LIN28 promotes protein synthesis and facilitates 2C gene repression during the development of pre-implantation embryo**s. (A) Schematic representation of siRNA microinjection experiments in (B), (C), (E–I) (B) qRT-PCR showing *Lin28a* mRNA level in blastocysts, after zygotic injection of siRNAs followed by *in vitro* culture of the injected embryos. A *Lin28a* siRNA pool was used to knock down the Lin28a gene in embryos and scrambled siRNA duplex was used as negative control (siNC). *P < 0.05, *t*-test, error bar: standard error of the mean. (C) Embryo immunostaining of LIN28A showing successful LIN28A knockdown. The image is representative of one of three independent experiments. Scale bar, 50 μm. (D) Blastocyst formation rate was determined after zygotic injection of *Lin28a* siRNA pool or control siRNA. (E) Fluorescence imaging showing OP-puromycin-labeled (1 h) embryos cultured *in vitro* at different stages. Scale bar, 50 μm. (F) OP-puromycin labeling (1 h) and LIN28A expression immunostaining in control and *Lin28*a knockdown morula stage embryos. Scale bar, 50 μm. *P < 0.05, *t*-test, error bar: standard error of the mean. (G) qRT-PCR showing the expression of 2C-related genes in blastocysts, after zygotic injection of siRNAs followed by *in vitro* culture of these embryos. *P < 0.05, **P < 0.01, ***P < 0.001, two-way ANOVA, *n* = 3, error bar: standard error of the mean. (H) Log_2_(fold change) of gene expression abundance between *Lin28a* knockdown and control embryos for each gene cluster described in Fig. 2B. *P < 0.05, ***P < 0.001, Mann-Whitney U test. (I) The expression of two sub-classes MERVL genes, MERVL-int and MT2_Mm, in *Lin28a* knockdown and control embryos. ***P < 0.001, Wilcox signed rank test. (J) GSEA analysis showing collective changes in Ribosome gene set in *Lin28a* knockdown and control embryos. NES: normalized enrichment score

We have revealed that LIN28 promotes rRNA synthesis as well as represses the 2C-like transcriptional program by *Dux* in cultured pluripotent stem cells. Subsequently, in order to better understand the physiological role of nucleolar LIN28 in the development of early embryos, we injected a pool of *Lin28a* siRNAs into fertilized single cell embryos (Fig. 7A). Upon successful knockdown of LIN28A (Fig. 7B and 7C), embryos can proceed to blastocyst stage (Fig. 7D), consistent with previous findings that *Lin28a* knockout mice can develop through embryogenesis, although they manifest a dwarfism phenotype at E13.5 (Shinoda et al., 2013). However, for the previous study, it was unclear from which day the developmental defects start to accumulate and ultimately lead to the dwarfism phenotype. Considering the LIN28A nucleolar localization at the pre-implantation stage, and its roles in regulating ribosome and translation described above, we determined the protein synthesis rate in embryos using OP-Puromycin staining. We selected the 3.5-day morula stage because protein synthesis in cultured embryos reached to the highest peak at this development stage (Fig. 7E). We observed that *Lin28a* knockdown significantly reduced protein synthesis at the morula stage (Fig. 7F). Importantly, higher expression of 2C genes such as *Zscan4d*, *Gm4340*, *GM12794*, *Dux* and ERV genes such as *MERVL-pol* was also found in knockdown embryos at the blastocyst stage (Fig. 7G). RNA-seq also demonstrated up-regulated 2C/4C cluster of genes and ERV genes (Fig. 7H and 7I), and down-regulated ribosomal genes (Fig. 7J) in *Lin28a* knockdown embryos compared with controls, consistent with the results from cultured ES cells described above (Figs. 2B–E and 4D). Together, these results support a critical role of LIN28 in both repressing 2C genes and promoting ribosomal genes during the transition from the 2-cell/4-cell to the morula/blastocyst stage embryos.

## DISCUSSION

During pre-implantation embryo development, multiple waves of genes are activated sequentially, most prominently the so-called 2C genes which include ERVs expressed at the 2-cell/4-cell stages (Jukam et al., 2017; Rodriguez-Terrones and Torres-Padilla, 2018). In cultured ES cells, 2C-like cells resemble 2-cell stage embryos in regards to their transcriptome and epigenome. Using this system, previous studies have provided mechanistic insights about how 2C genes are activated through key transcription factors such as *Dux* (De Iaco et al., 2017; Hendrickson et al., 2017; Whiddon et al., 2017), and repressed through SETDB1/TRIM28-mediated H3K9 trimethylation (Matsui et al., 2010; Macfarlan et al., 2012; Messerschmidt et al., 2012; Maksakova et al., 2013) or HDAC1/2-mediated histone deacetylation (Macfarlan et al., 2012). Additionally, other factors impacting epigenetic mechanisms in 2-cell like cells have also been reported, such as SUMO2-mediated sumoylation of TRIM28 (Yang et al., 2015), TET2 and its partner PSPC1-regulated epigenetic alterations (Guallar et al., 2018), CAF-1-mediated chromatin remodeling (Ishiuchi et al., 2015), ZMYM2/LSD-mediated ERV expression (Yang et al., 2020), etc. Here, we show that LIN28 is one of the first RNA binding proteins involved in 2C and ERV gene regulation. In early embryo development, global DNA demethylation after fertilization results in ERV activation, whereas ERV suppression occurs mainly through H3K9 methylation (Wang et al., 2018). LIN28A protein has been reported to directly interact with TET1 protein and DNA (Zeng et al., 2016). However, according to Zeng et al.’s study, loss of LIN28A would lead to decreased function of TET1 and DNA hypermethylation, which does not account for enhanced expression of activated 2C and ERV genes in the *Lin28a* knockout cells. Thus, LIN28 might not function through TET1 and DNA methylation to regulate ERV activation. In contrast, we observed elevated *Dux* and reduced H3K9me3 levels at the ERV regions in *Lin28* knockout cells. Also, analysis of LIN28A ChIP (Zeng et al., 2016) suggests LIN28A does not appear to have enrichment of direct binding to ERV or 2C gene loci.

Here, we propose a novel mechanism of nucleolar LIN28 in regulating the 2C program and the homeostasis between 2-cell like and ES cells (Fig. S7F). In the wild-type ES cell program, LIN28 in the nucleolus resides in the same complex with NCL/TRIM28 and FBL or in the liquid phase with other nucleolar RNAs/proteins and peri-nucleolar chromatin containing *Dux*, and it mediates NCL/TRIM28 occupancy on the *Dux* locus to repress *Dux* expression. Meanwhile, LIN28 also facilitates NCL to bind rDNA and rRNA to promote rRNA expression and ribosome biogenesis. In contrast, in the *Lin28* knockout ES cells, as the LIN28-mediated complex or liquid phase is interrupted, *Dux* repression is released and rRNA expression is reduced which both contribute to the 2-cell like cell program. Nucleolar stress also activates p53 which can directly bind to *Dux* locus and promotes *Dux* expression.

LIN28 is traditionally known for its function in regulating *let-7* in both the cytosol and the nucleus through blocking DICER and DROSHA complexes, respectively (Viswanathan and Daley, 2010; Piskounova et al., 2011). Recent studies have also shown that it binds to numerous mRNAs and influences RNA metabolism and translation primarily in the cytosol (Cho et al., 2012; Wilbert et al., 2012). In contrast to the localization pattern observed in certain cancer cells, in which LIN28B is more likely to be detected in the nucleus whereas LIN28A localizes to the cytosol (Piskounova et al., 2011), the two paralog proteins appear to localize both in the nucleolus and the cytosol in pluripotent stem cells. Although LIN28 has putative nucleolar localization sequences (Piskounova et al., 2011; Kim et al., 2014), its nucleolar functions have not been elucidated before.

We found that in the nucleolus of mouse naïve ES cells, LIN28A interacts with snoRNA and rRNA. Recently, it has become clear that RNA and RNA binding proteins form membrane-less organelles, or multilayered phase-separated ribonucleoprotein bodies such as nucleoli (Falahati et al., 2016; Feric et al., 2016; Langdon and Gladfelter, 2018). It appears that these nucleolar RNA species, together with the LIN28 protein and nucleolar/ribosomal proteins form a phase-separated nucleolar sub-compartment for their functions in unperturbed conditions. Analogous to the role of LIN28 on pri- and pre-microRNA *let-7* (Viswanathan et al., 2008; Heo et al., 2009; Nam et al., 2011), LIN28 might directly or indirectly involved in rRNA processing and maturation. Even though the detailed LIN28/snoRNA or LIN28/rRNA complex structures remain unknown, we observed that LIN28-deficient PSCs had decreased levels of rRNA whose maturation is dependent on snoRNA-mediated modifications (Kiss, 2002) or NCL/FBL-mediated processing (Ginisty et al., 1998). This rRNA de-regulation can lead to: i) decreased protein synthesis which was suggested as a feature of 2C-like cells (Hung et al., 2013; Eckersley-Maslin et al., 2016), and ii) nucleolar stress and activation of p53 signaling pathway (Zhang and Lu, 2009; Golomb et al., 2014). The mechanism leading to p53 stabilization involves disruption of the ribosome large unit, release of free ribosomal proteins such as RPL5, RPL11 and RPL23 into the cytosol, and RPL5 binding to and sequestration of MDM2 protein to prevent p53 ubiquitination and degradation (Lohrum et al., 2003; Dai and Lu, 2004; Dai et al., 2004). In our study we observed a global de-regulation of ribosomal gene expression and of ribosomal protein localization, accompanied by an increase of p53 and activation of 2C genes. Beyond the cause of genetic ablation of LIN28, RNA Pol I inhibitor treatment also caused nucleolar stress characterized by reduced rRNA levels and altered nucleolar morphology. Exposure to other types of stress such as DNA damage, oxidative stress in ES cell cultures can also indirectly interfere with nucleolar function and cause nucleolar stress (Rubbi and Milner, 2003; Yang et al., 2016; Yang et al., 2018), and our findings open up the possibility that ribosomal and nucleolar stress are a common denominator for variation of ERV expression often observed in ES cell cultures.

Not only can LIN28 pull down rRNA, snoRNA and ribosomal proteins, it also interacts with NCL/TRIM28 complex in an RNA-dependent manner. NCL is a DNA and RNA binding protein required for rRNA synthesis and pre-RNA processing, and recent studies have shown its more versatile roles in chromatin remodeling (Mongelard and Bouvet, 2007; Jia et al., 2017) and in repressing 2C program in ES cells through a complex containing TRIM28 and LINE1 RNA (Percharde et al., 2018). LIN28 appears to reside in and be required for forming this nucleolar sub-compartment with NCL/TRIM28 complex organized around the *Dux* loci at the peri-nucleolar region. This compartment may also be an attractive region of nuclear heterochromatin (Guetg and Santoro, 2012). Loss of LIN28 may lead to disruption of this compartment, reduced occupancy of NCL/TRIM28 complex on *Dux* and rDNA loci, and consequently lead to de-repression of *Dux* and ERV genes mediated by TRIM28, and reduced expression of rRNA mediated by NCL. Interestingly, a nuclear m6A reader YTHDC1 regulates the function of nuclear LINE1 scaffold and promotes the formation of the LINE1-NCL-KAP1 complex (Chen et al., 2021), however, we did not find a significant enrichment of direct LIN28A binding to the RNAs of LINE1 in global levels.

LIN28’s dual roles in both repressing 2C genes and promoting ribosomal genes expression is in line with the reported dual roles of NCL/TRIM28 (Percharde et al., 2018). As a matter of fact, embryo development is a series of tightly-regulated transitions through different stages, and whether a factor can simultaneously shut down an earlier program, such as the 2-cell/4-cell program, and initiate a later stage program, such as the 4-cell/8-cell program, is an interesting question. Like LINE1/Nucleolin/TRIM28 complex, we propose that LIN28 is a crucial factor to bridge two adjacent processes after ZGA: on one hand, LIN28 shuts down the 2-cell/4-cell program, and on the other hand, LIN28 promotes a pro-proliferation and anabolic program and a shift away from the 2C-like state with a metabolically inactive and translationally inert program. Taken together, our data provide a new perspective that ending the 2C/4C program is not a passive process, but instead it is actively facilitated by the concomitant ensuring ribosomal gene expression and nucleolar maturation at the 4C/8C stage whereby LIN28 plays a critical role. Future works on ribosomal and nucleolar proteins will help us to have a more comprehensive understanding of the roles of ribosome/nucleolus in coordinating anabolic growth and proliferation, stress signal transduction and the responsive gene regulation (Boulon et al., 2010) in the context of pluripotent stem cells and early embryo development.

## MATERIALS AND METHODS

### Cell culture

Mouse ES cells E14 (or V6.5 when indicated) and iPS cells were cultured on 0.1% gelatin-coated plates with MEF feeder cells in N2/B27/LIF/2i media (1:1 mix of DMEM/F12 (11320-033, Gibco) and Neurobasal medium (21103-049, Gibco) containing 1× N2 and B27 supplements (17502-048/17504-044, Life Technologies), 100 µmol/L non-essential amino acids (GNM71450, GENOM), and 1,000 U/mL LIF (PEPRO TECH), 1 μmol/L PD03259010 and 3 μmol/L CHIR99021 (STEMCELL Technologies) and 100 U/mL penicillin, 100 µg/mL streptomycin (15140-122, Gibco). For primed state media, 20 ng/mL Activin, 10 ng/mL FGF2, and 1% KSR were added to the 1:1 DMEM/F12 and Neurobasal medium containing N2 and B27. MEF cells were cultured in high-glucose DMEM (11960-069, Gibco) containing 10% FBS (10099-141, Gibco), 100 µmol/L nonessential amino acids (GNM71450, GENOM) and 100 U/mL penicillin, 100 µg/mL streptomycin (15140-122, Gibco).

### Mouse embryo collection and culture

Embryos were derived from 4- to 6-week-old C57BL/6J (C57) females mated to 8-week to 3-month old C57BL/6J (C57) males. Females were superovulated by injection of 7.5 IU PMSG, followed by injection of 7.5 IU of hCG 46–48 h later. Each stage of embryos were collected at the following time periods: 20–24 h after hCG (zygotes), 31–32 h (early 2-cell), 39–40 h (mid 2-cell), 46–48 h (late 2-cell), 54–56 h (4-cell), 68–70 h (8-cell), 76–78 h (16-cell), 86–88 h (early blastocysts), 92–94 h (mid blastocysts), and 100–102 h (late blastocysts) (Deng et al., 2014). Zygotes were collected from ampullae of oviducts and released into a hyaluronidase (Sigma-Aldrich)/M2 (Sigma-Aldrich) solution for removing cumulus cells. Cleavage stage embryos were flushed from oviducts and uterus in M2 medium, blastocysts were flushed from the uteri. For *in vitro* culture, an embryo was washed through 3 drops of M2 and then cultured in a 20 µL drop of KSOM medium (Millipore) in mineral oil (Sigma-Aldrich) at 37 °C, 5% CO_2_. All animal care and experimental procedures were in accordance with the Animal Research Committee guidelines of Zhejiang University.

### *2C::tdTomato* mES cell lines

The *2C::tdTomato* cells were generated as in (Macfarlan et al., 2012). mES or miPS cells were transfected with *2C::tdTomato* using Lipofectamine 2000 and selected with 150 μg/mL hygromycin 48 h after transfection and for 7 days.

### Generation of *Lin28a* knockout ES cell lines with CRISPR/CAS9

sgRNA was designed to target the second exon of *Lin28a* using the online tool: (https://portals.broadinstitute.org/gpp/public/analysis-tools/sgrna-design), then cloned into the gRNA-Cas9-Puro Plasmid (L00691, GenScript). ESCs were nucleofected with the plasmid containing the sgRNA and Cas9 using Lonza 4D Nucleofector. 48 h after transfection ESCs were selected with 1 μg/mL puromycin for 7 days. Clones were picked and analyzed for LIN28A expression by Western blot, and the *Lin28a* gRNA targeted genomic region was PCR-amplified and sequenced in *Lin28a* knockout clones.

### siRNA-mediated knockdown in PS cell lines

siRNA transfections were performed in PS cells with Lipofectamine 2000 (Thermo Fisher Scientific). PS cells were seeded into 12-well plate and cultured in LIF/2i medium for overnight. The next day, 800 μL LIF/2i medium without antibiotics was added into each well. Then, the transfect mixture (40 pmol of 3 independent siRNA targeting each gene/a non-targeting siRNA (negative control, NC) and 2 μL of Lipo 2000 which was diluted in 200 μL Opti-MEM medium (Gibco)) was added into each well and incubated for 6 h at 37 °C. After incubation, the medium was exchanged for fresh complete LIF/2i medium and cells were harvested for RNA extraction approximately 48 h later. The sequences of siRNA are listed in Table S3.

### Embryo microinjection of siRNA

The *Lin28a* pooled siRNA and non-targeting pooled siRNA (Dharmacon Germany) were resuspended in RNase free water according to the manufacturer’s instructions and stored in single-use aliquots at −80 °C. siRNAs were microinjected using an Eppendorf FemtoJet microinjector and Narishige micromanipulators. Microinjection pipettes were pulled with a Sutter P-97 pipette puller. siRNA solution (2 μL of 50 μmol/L) was loaded into the pipette and ~5 pL was injected into the cytoplasm of each zygote. A relatively consistent amount was carefully injected each time. After injection, zygotes were rinsed and cultured at 37 °C with 5% CO_2_. The sequences of siRNA used are listed in Table S3.

### Cell line immunofluorescence staining

ES cells were plated on gelatinized glass coverslips on primary mouse embryonic fibroblasts. Cells were fixed with 4% PFA for 30 min followed by permeabilizing with 0.5% Triton X-100/PBS for 20 min at RT. Cells were blocked in blocking buffer (3% BSA, 2% donkey serum in PBS) for 10 min and stained with a primary antibody for 1 h at 37 °C. After washing three times for 10 min with PBS, cells were stained with a secondary antibody for 1 h at 37 °C. Antibodies used: For LIN28A (rabbit, Cell Signaling), a rabbit antibody at 1:300 and a donkey anti-rabbit Alexa Fluor 488-conjugated secondary antibody (abcam) at 1:400 were used. For Fibrillarin (mouse, abcam) and NPM1 (mouse, Sigma-Aldrich), the mouse antibodies at 1:200 and a DyLight 594 goat anti-mouse secondary antibody (EarthOx) at 1:400 were used. Following washing for another three times with PBS, DAPI was used for nucleus staining. Cells were then imaged using Zeiss LSM880 fluorescence microscope at a 63× oil objective. For high regulation microscopy imaging, LSM800 with Airyscan module was used.

### Fluorescence recovery after photobleaching (FRAP) analysis

Mouse E14 wild-type and knockout ES cells cultured on MEF cells were grown in N2B27/LIF/2i conditions and maintained at 37 °C and with 5% CO_2_ during image acquisition. Cells were transduced with pSIN-FBL-mCherry lentivirus. FRAP experiments were performed on a ZEISS (Jena, Germany) LSM800 confocal laser scanning microscope equipped with a ZEISS Plan-APO 63×/NA1.46 oil immersion objective. Circular regions of constant size were bleached and monitored overtime for fluorescence recovery. Bleaching was once every 10 s for a total of 10 min. Fluorescence intensity data were corrected for background fluorescence, and normalized to initial intensity before bleaching using GraphPad software. Resulting FRAP curves were fitted with Four parameter logistic (4PL) curve.

### Embryo immunofluorescence staining

Embryos were first fixed with 1% and 2% paraformaldehyde (PFA) in 1× PBS for 3 min sequentially, followed by treatment with 4% PFA for 30 min at room temperature (RT). Embryos were washed three times with 1× PBS, permeabilized for 15 min in PBS/0.25% Triton X-100 and blocked in blocking buffer (PBS/0.2% BSA/0.01% Tween-20) for 1 h at RT, followed by incubation overnight with primary antibodies (LIN28A, Cell Signaling, 1:50; Fibrillarin, Abcam, 1:100; GAG, Epigentek, 1:200) at 4 degree or for 1 h at 37 °C. Subsequently, embryos were washed four times for 10 min each and incubated with a secondary antibody (daylight 488-conjugated anti-rabbit, 1:100 or daylight 594-conjugated anti-mouse, 1:200) for 1hr at 37 °C and washed three times with PBS. Nuclei were stained with DAPI for 1 min. Embryos were observed under Zeiss LSM880 fluorescence microscope at 63× magnification with an oil immersion objective.

### Cell line RNA extraction and quantitative PCR

Total RNA was isolated from ESCs and miPSCs using miRNeasy kit (217004, QIAGEN) according to the manufacturer’s protocol, and 1 μg RNA was reverse transcribed to cDNA with HiScript II Q RT Super Mix (R223-01, Vazyme). Gene expression was analyzed with SYBR-Green qPCR Master mix (Bio-Rad) on Bio-Rad PCR machine (CFX-96 Touch). Each gene was normalized to *Actin* or *Gapdh*. For microRNA expression analysis, reverse transcription and qPCR were performed with miRNA cDNA synthesis kit and qPCR assay kit (CW2141/CW2142, CWBIO), each gene was normalized to U6. All primers used are listed in Table S2.

### Embryo collection, cDNA synthesis and real-time PCR

Ten morulae were rinsed in 0.2% BSA/PBS without Ca^2+^ and Mg^2+^ and placed in 0.2 Ml PCR tube, immediately transferred in liquid nitrogen and stored at −80 °C. It was hybridized with 0.5 µL oligo-dT30 (10 μmol/L, Takara) and 1 μL random (1 mol/L) and 1 µL dNTP mix (10 mmol/L) in 2 μL cell lysis buffer (2 U RNase inhibitor, 0.01% Triton X-100) at 72 °C for 3 min. Then, the reaction was immediately quenched on ice. After the reaction tube was centrifuged, 2 µL was used for reverse transcription with Super Script II Reverse Transcriptase 5× first strand buffer, 0.25 µL RNase inhibitor (40 U), 0.06 µL MgCl_2_ (1 mol/L), 2 µL betaine (5 mol/L), and 0.5 µL Reverse Transcriptase Superscript II (Takara). Reverse transcription was carried out in the thermocycler at 42 °C for 90 min, 70 °C for 15 min, and then 4 °C for holding. Subsequently, cDNA was diluted 1:10 (*v*/*v*) with RNase free water and used for a qPCR amplification in triplicate with SYBR Green Master (Vazyme) in a final volume of 20 µL per reaction as manufacturer’s instructions. The primers used are listed in Table S2.

### Cell line RNA-seq library preparation and sequencing

A total amount of 2 μg RNA per sample was used as input materials for the RNA sample preparation. mRNA was purified from total RNA using poly-T oligo-attached magnetic beads. Purified mRNA was fragmentated at 94 °C for 15 min by using divalent cations under elevated temperature in NEBNext first strand synthesis reaction buffer (5×). First strand cDNA was synthesized using random primer and ProtoScript II reverse transcriptase in a preheated thermal cycler as follows: 10 min at 25 °C; 15 min at 42 °C; 15 min at 70 °C. Immediately finished, second strand synthesis reaction was performed by using second strand synthesis reaction buffer (10×) and enzyme mix at 16 °C for 1 h. The library fragments were purified with QiaQuick PCR kits and elution with EB buffer, then terminal repair, A-tailing and adapter added were implemented. The products were retrieved and PCR was performed for library enrichment. The libraries were sequenced on an Illumina platform.

### Embryo RNA-seq library preparation and sequencing

Embryos were collected (5 embryos per sample) in 0.2 mL PCR tubes with a micro-capillary pipette and processed into cDNA with Superscript II reverse transcriptase as described described previously (Picelli et al., 2014). The cDNA is amplified with KAPA Hifi HotStart using 12 cycles. Sequencing libraries were constructed from 1 ng of pre-amplified cDNA using DNA library preparation kit (TruePrep DNA Library Prep Kit V2 for Illumina, Vazyme). Libraries were sequenced on a HiSeq-PE150, with paired end reads of 150 bp length each.

### Overexpression construct transfection

Overexpression vector were introduced into cells by using 4D Nucleofector (Lonza). 1 × 10^5^ ES cells were used per nucleofection together with 5 μg vector. Immediately after transfection, cells were plated in LIF/2i medium and in culture for 48 h. Then, cells were harvested for protein/RNA extraction.

### Cell cycle analysis

PS cells were seeded into 6-well plates and cultured for 48 h. The cells were harvested and cell suspensions were pelleted and washed twice with PBS, and fixed in 70% ice-cold ethanol overnight at −20 °C. The samples were centrifuged at 1,100 rpm for 2 min at 4 °C. The pellets obtained were washed twice with cold PBS and treated with 100 μg/mL RNase A for 10 min at 37 °C. Cellular DNA was stained with 10 μg/mL propidium iodide (PI) for 20 min at 37 °C in darkness, and the cell cycle distribution were analyzed with flow cytometry (FC500MCL, Beckman) and FlowJo software.

### Co-immunoprecipitation for protein-protein interaction

The PS cells were lysed with RIPA lysis buffer containing protease-inhibitors for 30 min on ice and centrifuged. Cell lysate was combined with 2.5 μg of IP antibody (anti-LIN28A, normal IgG) per sample and incubated with rotating overnight at 4 °C. Next day, the mixture was transferred to 50 μL pre-washed magnetic beads and incubated with rotating for 3 h at room temperature. Beads were collected with a magnetic stand and washed at least 3 times, followed by addition of 40 μL 1× SDS-PAGE loading buffer to the complex and incubation at RT for 15 min with mixing. Beads were magnetically separated and the supernatant was saved for following analysis.

### Western blot

The ES and iPS cells were lysed with RIPA lysis buffer containing protease-inhibitors for 30 min on ice and centrifuged at 13,000 rpm for 15 min, and then the supernatant was transferred to new tubes carefully. Protein concentration was quantified using BCA protein assay kit (P0012, Beyotime), 20 μg denatured protein samples were separated by 12% SDS-PAGE and transferred onto PVDF membranes. Blocking was performed for 1 h in 5% non-fat milk/PBS-T buffer followed by incubation overnight with primary antibodies at 4 °C. The next day, membranes were incubated with the appropriate secondary antibodies conjugated to HRP for 1 h at room temperature, and the bands were detected by ECL reagent and autoradiography.

### Chromatin Immunoprecipitation (ChIP)

We added 9 mL fresh cell culture medium containing formaldehyde to a final concentration of 1% to cells for 10 min at 37 °C to crosslink. Then, we added 1 mL of 1.25 mol/L glycine into 9 mL of cross-link solution and incubated at room temperature for 5 min, ice-cold PBS wash cells twice. Cell pellet was resuspended with lysis buffer containing 1× Protease Inhibitor Cocktail and incubated on ice for 10 min. Afterwards, the chromatin lysate was transferred to a 1.5 mL centrifuge tube and chromatin sheared using water bath sonication with the following conditions: shear 15 cycles at 4 °C, 15 s on, 30 s off. Centrifuge and transfer supernatant to a new tube. For input, take 5 μL (1%) from the 500 μL containing sheared chromatin. Each chromatin sample was incubated with antibodies for H3K9me3 (ab8898, abcam), NCL (ab22758, abcam), TRIM28 (ab22553, abcam) or normal IgG (2729, CST) overnight on a rotating platform at 4 °C. The next day, the sample was incubated with protein A + G magnetic beads (HY-K0202, MCE) for 2 h at 4 °C with rotation. The beads-antibody/chromatin complex was washed with wash buffer and resuspended with elution buffer. Then the elute DNA was treated with RNase A at 42 °C for 30 min, then treated with protease K at 60 °C for 45 min followed by heat inactivation at 95 °C for 15 min. The purified DNA was subjected to library preparation or analyzed by qPCR. The primers used are listed in Table S2.

### ATAC-seq library preparation and sequencing

50,000 cells (maximum) were harvested and lysed in 50 μL of cold lysis buffer (10 mmol/L Tris-HCl (pH 7.4), 10 mmol/L NaCl, 3 mmol/L MgCl2, 0.1% (*v*/*v*) Igepal CA-630) for 10 min on ice to prepare the nuclei. Then, spin down immediately at 500 ×*g* for 5 min, 4 °C, to remove the supernatant. Nuclei were then incubated with TruePrep Tagment Enzyme (TD501, Vazyme) at 55 °C for 10 min. Immediately after the tag mentation, 1× VAHTS DNA Clean Beads (N411, Vazyme) was added into the reaction to purify DNA fragments. PCR amplify the library with the following program: 72 °C for 3 min; 98 °C for 30 s; and thermocycling at 1 cycle of 98 °C for 15 s, 15 cycles of 60 °C for 30 s and 72 °C for 3 min; following by 72 °C for 5 min. Libraries were purified with the 0.6×/0.15× double sided size selection. Libraries were sequenced with the Illumina HiSeq 2500 System.

### RNA immunoprecipitation

The cytoplasmic and nuclear extracts were separated and prepared from PS cells according to a standard Thermo protocol (78833). 0.5 µg of the IP antibody (anti-LIN28A, ab63740, abcam; FBL, ab4566, abcam; normal mouse IgG, PP64B, Merck) were pre-bound to 50 µL of Magnetic Protein A/G (Merck) and incubated with rotation for 30 min at room temperature. 100 µL of cytoplasmic and nuclear extracts were removed and added to each beads-anti-body complex in the presence of proteinase and RNase inhibitors, followed by incubation with rotating for 3 h at 4 °C. After washing, each immunocomplex was re-suspended in 150 µL of proteinase K buffer and incubated at 55 °C for 30 min with shaking to digest the protein. Then RNA was extracted with TRIzol for qRT-PCR.

### OP-Puro labeling

To measure protein synthesis, PS cells or mouse embryos were cultured for 1 h in 2i medium or EmbryoMax KSOM medium + amino acids (Merck Millipore) respectively, and labeling experiments were performed using Click-iT® Plus OPP Protein Synthesis Assay Kit (Life Technologies, C10456). After labeling, the samples were fixed for 15 min at room temperature in PBS supplemented with 4% paraformaldehyde and then permeabilized in PBS supplemented with 0.25% Triton X-100 for 15 min at room temperature according to the manufacturer’s instructions. Nuclei were stained with DAPI for 2 min and the cell were observed under Zeiss LSM880 fluorescence microscope. For flow cytometry, PS cells were cultured for 1 h in 2i medium supplemented with component A (Click-iT® Plus OPP Protein Synthesis Assay Kit, Life Technologies, C10456) and then were fixed for 15 min in 4% PFA at room temperature.

### Transmission electron microscope (TEM)

For transmission electron microscopy, PS cells cultured in one 10 cm dish were collected, cleaned from feeder cells and supplemented with 2.5% glutaraldehyde. The cell pellet was dispersed into small clusters and fixed at least 6 h at 4 °C. After that, the cell samples were treated with standard procedures. Then the slices were imaged on FEI Spirit 120 kV LaB6 Routine Cryo-EM Capable Electron Microscope.

### Quantification of 2’-O-Me residues in RNA using next-generation sequencing (RiboMeth-seq) (Birkedal et al., 2015)

First, total RNA (2 µg) was subjected to the metal ion-based RNA cleavage (magnesium or zinc-ions). RNA hydrolysis was performed in the 100 mmol/L Tris-Cl buffer, pH 8.0, containing either 0.1 mmol/L ZnCl_2_ or 2 mmol/L MgCl_2_ for 2 or 3 min at 95 °C. The reaction was stopped by ethanol precipitation with 3 mol/L Na-OAc, pH 5.2. In addition, the zinc-based RNA cleavage was stopped by adding 2 µL of 0.5 mol/L EDTA pH 8.0 before ethanol precipitation. After centrifugation, the pellet was washed with 80% ethanol and then was resuspended in nuclease-free water. The sizes of generated RNA fragments were assessed by capillary electrophoresis using a PicoRNA chip on Bioanalyzer 2100 (Agilent, USA) and its range is from 30 to 200 nt. RNA fragments were converted to library using VAHTS Small RNA Library Prep Kit for Illumina (NR801-01), following the manufacturer’s instructions. DNA library quality was assessed using a high sensitivity DNA chip on a Bioanalyzer 2100. Library quantification was done using a fluorometer (Qubit2.0 fluorometer, Invitrogen, USA).

### Bulk and single-cell RNA-seq data analysis

All bulk RNA-seq reads were trimmed using Trimmomatic software with the following parameters “ILLUMINACLIP:TruSeq3-PE.fa:2:30:10 LEADING:3 TRAILING:3 SLIDINGWINDOW:4:15 MINLEN:36” (Version 0.36) (Bolger et al., 2014). and were further quality-filtered using FASTX Toolkit’s fastq_quality_trimmer command (Version 0.0.13, http://hannonlab.cshl.edu/fastx_toolkit/) with the minimum quality score 20 and minimum percent of 80% bases that has a quality score larger than this cutoff value. The high-quality reads were mapped to the mm10 genome by HISAT2, a fast and sensitive spliced alignment program for mapping RNA-seq reads, with -dta paramenter (Daehwan et al., 2015). PCR duplicate reads were removed using Picard tools and only uniquely mapped reads were kept for further analysis. The expression levels of genes and repeat sequences were independently calculated by StringTie (Mihaela et al., 2015) (Version v1.3.4d) with -e -B -G parameters using Release M18 (GRCm38.p6) gene annotations downloaded from GENCODE data portal and annotated repeats (RepeatMasker) downloaded from the UCSC genome browser, respectively. To obtain reliable and cross-sample comparable expression abundance estimation for each gene and each family of repeat sequence, reads mapped to mm10 were counted as TPM (Transcripts Per Million reads) based on their genome locations. Differential expression analysis of genes in different samples was performed by DESeq2 using the reads count matrix produced from a python script “prepDE.py” provided in StringTie website (http://ccb.jhu.edu/software/stringtie/). The stage-specific scores of genes expressed during mouse early embryo development were obtained by entropy-based measure (Cabili et al., 2011). We then selected the genes with stage-specific scores larger than 0.2 to perform K-mean clustering analysis using pheatmap R package.

For single cell RNA-seq, we used InDrop system following the manufacturer’s protocol (OneCell). We followed the previously published pipeline (Zilionis et al., 2016) to product digital gene expression matrices of the droplet microfluidics-based single-cell RNA-seq sequencing data derived from wild-type (a2.1) and *Lin28a* knockout (a3.5) mouse ES cells. Single-cell gene expression matrix was further analyzed with Seurat v2.3.4 (https://satijalab.org/seurat/). We excluded the genes with expressed cell number smaller than 3 and the cells with nUMIs smaller than 500 or the expression percentages of mitochondrial genes larger than 0.2 and used 16 Principle Components (PCs) for tSNE analysis. All other bulk and single-cell RNA-seq data analyses and visualization were performed in R/Bioconductor utilizing custom R scripts.

### Crosslinking-immunoprecipitation sequencing (CLIP-seq) data analysis

For public CLIP-seq data (Cho et al., 2012), we first downloaded the SRA files from NCBI Sequence Read Archive (SRA). After the SRA files were gathered, the archives were extracted and saved in FASTQ format using SRA Toolkit. We followed the trimming and quality-checking procedure that we used for bulk RNA-seq data processing. Subsequently, the CLIP-seq reads were aligned to mouse ribosomal DNA complete repeating unit (GenBank accession BK000964.1) by Bowtie with the parameters “-n 0 -norc –best -l 15”. Filtered reads that do not match to mouse ribosomal DNA complete repeating unit were mapped to mm10 genome by HISAT2 with -dta paramenter. The alignment results were filtered to remove PCR duplicate reads and leave only the uniquely mapped reads. The CLIP tag density at the transcript level was qualified by TPM value of mapped CLIP-seq reads of a given region (defined by GENCODE M18 gene annotation or RepeatMasker repeat sequence annotation) produced from StringTie with -e -B -G parameters. The CLIP tag enrichment at the transcript level was estimated as log2 ratio between the TPM value from the CLIP library and the TPM value from the control RNA-seq library.

For in-house CLIP-seq data, we first trimmed 6bp random sequence of read’s 3’-terminal using fastx_trimmer command contained in FASTX Toolkit. Then, we clipped the adaptor sequence “AGATCGGAAGAGCACACGTCT” using FASTX Toolkit fastx_clipper. We used the same procedure as the public CLIP-seq data for subsequent analysis. The “bedtools genomecov” command with the parameters “-bg -dz -split” was used to produce bedgraph files for visualizing the CLIP-seq and RNA-seq signal in the UCSC genome browser. All other downstream CLIP-seq analyses and visualization were performed in R/Bioconductor utilizing in-house custom scripts.

### ATAC-seq and ChIP-seq data processing and analysis

To tailor and filter ATAC-seq and ChIP-seq reads, we used the same procedure as RNA-seq reads processing. For each sample, the retained ATAC-seq and ChIP-seq reads were first aligned to mm10 genomes using Bowtie2 (version 2.3.4.1). The ATAC-seq reads were aligned with the parameters: -t -q -N 1 -L 25 -X 2000 no-mixed no-discordant. The ChIP-seq reads were aligned to mm10 with the options: -t -q -N 1 -L 25. All unmapped reads and PCR duplicates were removed. The bamCoverage and bamCompare commands contained in deepTools (version 2.5.3) were adopted for downstream analysis. Using BamCoverage command with the parameters: -normalizeUsing BPM -of bigwig -binSize 100, we first normalized the raw reads signal to Bins Per Million mapped reads (BPM) signal and converted the alignment bam files to bigwig signal files. The bigwig files were imported into UCSC genome browser for visualization. To minimize the effect of chromatin structure and sequencing bias in our H3K9me3 ChIP-seq data, we corrected ChIP-seq signal using log2 ratio transformation between H3K9me3 signal and input signal by BamCompare command. The “computeMatrix” and “plotProfile” commands of deepTools were used to produce the reads density distribution plot of ATAC-seq and ChIP-seq signal in the given genomic region. For analyzing and visualizing public 5hmC-seq and MeDIP-seq data (Zeng et al., 2016), we adopted the same procedure as ATAC-seq data processing.

### RiboMeth-seq data processing and analysis

Initial trimming of adapter sequence was done using Trimmomatic-0.36 as described above. The 28s, 18s and 5s rRNA sequences were downloaded from NCBI Gene database. Alignment to these reference rRNA sequences was done by Bowtie2 (ver 2.2.4) with end-to-end mode and k = 1. Simultaneous 5′-end and 3′-end reads counting was done by bedtools v2.25.0 after conversion to *.bed fle. Final analysis was performed by calculating MethScore for single-base level quantification by using the method mentioned in (Birkedal et al., 2015; Marchand et al., 2016b).

### Statistical analysis

All statistical analyses for next generation sequencing (NGS) data were performed with R software. The other statistical analyses were performed with GraphPad Prism software. Details of individual tests are outlined within each figure legend, including number of replication performed (*n*) and the reported error as standard error of the mean (s.e.m). All statistics are *P < 0.05, **P < 0.01, ***P < 0.001, and were calculated by Wilcox signed rank test (for paired samples), Mann-Whitney U test (for independent samples), two-way ANOVA and *t*-test as described in the figure legends.

## Supplementary Information

Below is the link to the electronic supplementary material.

Supplementary file 1 (PDF 5238 kb)

Supplementary file 2 (XLSX 1229 kb)

Supplementary file 3 (XLSX 13 kb)

Supplementary material 4 (XLSX 9 kb)
