# Supplementary material for: LIN28 coordinately promotes nucleolar/ribosomal functions and represses the 2C-like transcriptional program in pluripotent stem cells
Source: Protein Cell. 2021 Jul 31;13(7):490–512. doi: 10.1007/s13238-021-00864-5 (PMC9226220; doi:10.1007/s13238-021-00864-5)
Supplement: Supplementary file 1 — (PDF 5238 kb) [file 13238_2021_864_MOESM1_ESM.pdf]

Supplementary Figures for

LIN28 Coordinately Promotes Nucleolar/Ribosomal Functions and Represses the 2C-like Transcriptional Program in Pluripotent Stem Cells

Zhen Sun[1, #], Hua Yu[1, #], Jing Zhao[1, #], Tianyu Tan[1], Hongru Pan[1], Yuqing Zhu[1], Lang Chen[1], Cheng Zhang[2], Li Zhang[1], Anhua Lei[1], Yuyan Xu[1], Xianju Bi[3], Xin Huang[4], Cristina Correia[2], Ming Chen[5], Qiming Sun[6], Li Shen[7], Jianlong Wang[4], Xiaohua Shen[3], George Q. Daley[8], Hu Li[2], Jin Zhang[1,9*]

Figure S1

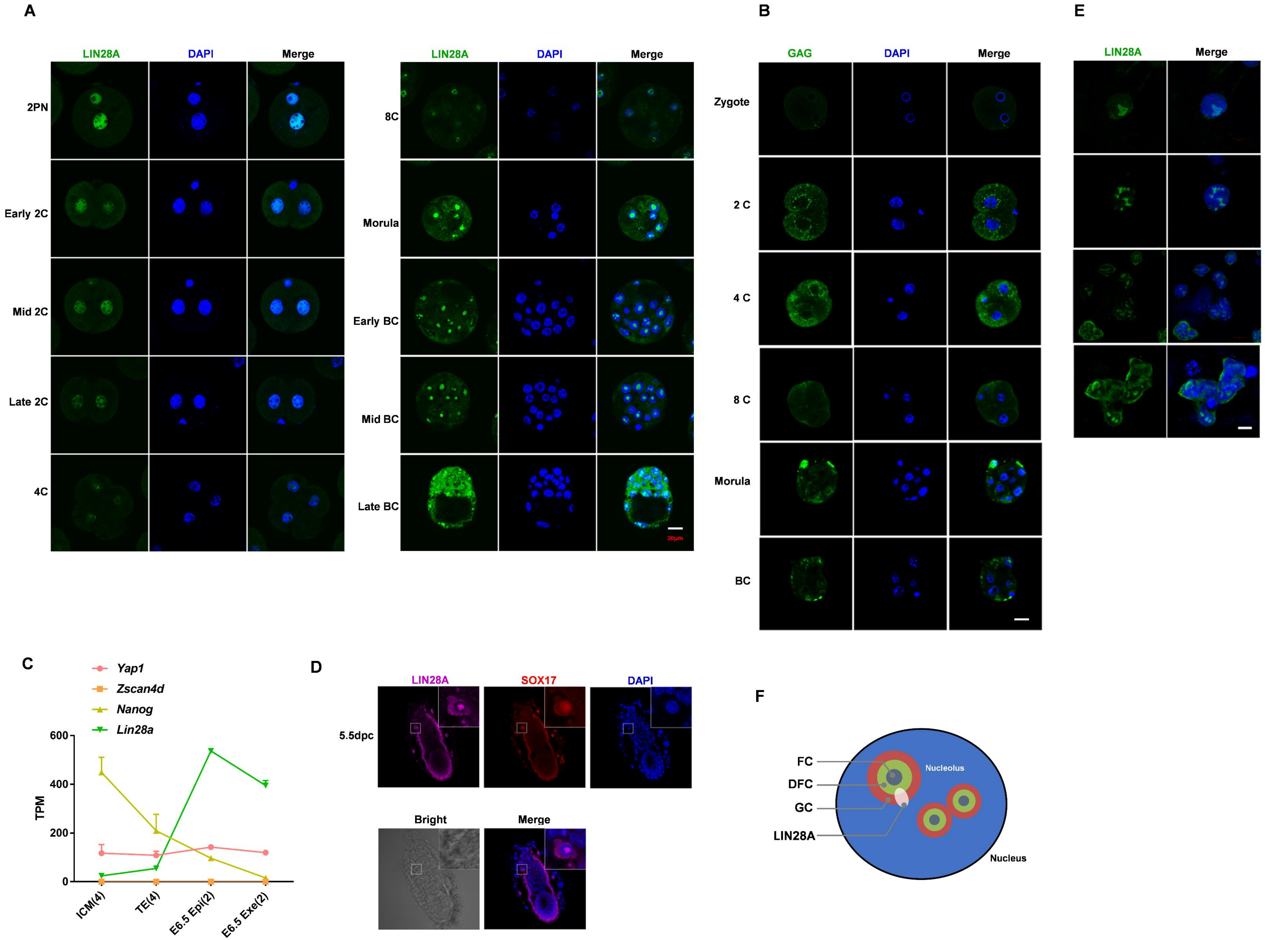
A
LIN28A
DAPI
Merge
2PN
Early 2C
Mid 2C
Late 2C
4C
8C
Morula
Early BC
Mid BC
Late BC
B
GAG
Zygote
2 C
4 C
8 C
BC
E
C
Yap1
Zscan4d
Nanog
Lin28a
TPM
600
400
200
0
ICM(4)
TE(4)
E6.5 Epi(2)
E6.5 Exe(2)
D
SOX17
5.5dpc
Bright
F
FC
DFC
GC
Nucleolus
Nucleus

Fig. S1. Temporal and spatial expression of LIN28A in mouse early embryo development. Related to Fig. 1.

(A) LIN28A immunostaining at indicated differentiation stages. Scale bar, 20 µm.

(B) Gag protein expression assessed by immunostaining during mouse early embryo development. Scale bar, 20 µm.

(C) Lin28a mRNA expression in post-implantation embryos analyzed from published RNA-seq data (Wang, et. al., 2018). Numbers in the parentheses mean the number of embryos.

(D) Immunostaining showing LIN28A protein expression in post-implantation embryos.

(E) Lin28A immunostaining in single pluripotent stem cell and small colonies. Scale bar, 10 µm.

(F) Cartoon illustrating three nucleolar compartments and Lin28A's localization in the nucleus. GC (granular component), DFC (dense fibrillar component), and FC (fibrillar center).

Figure S2

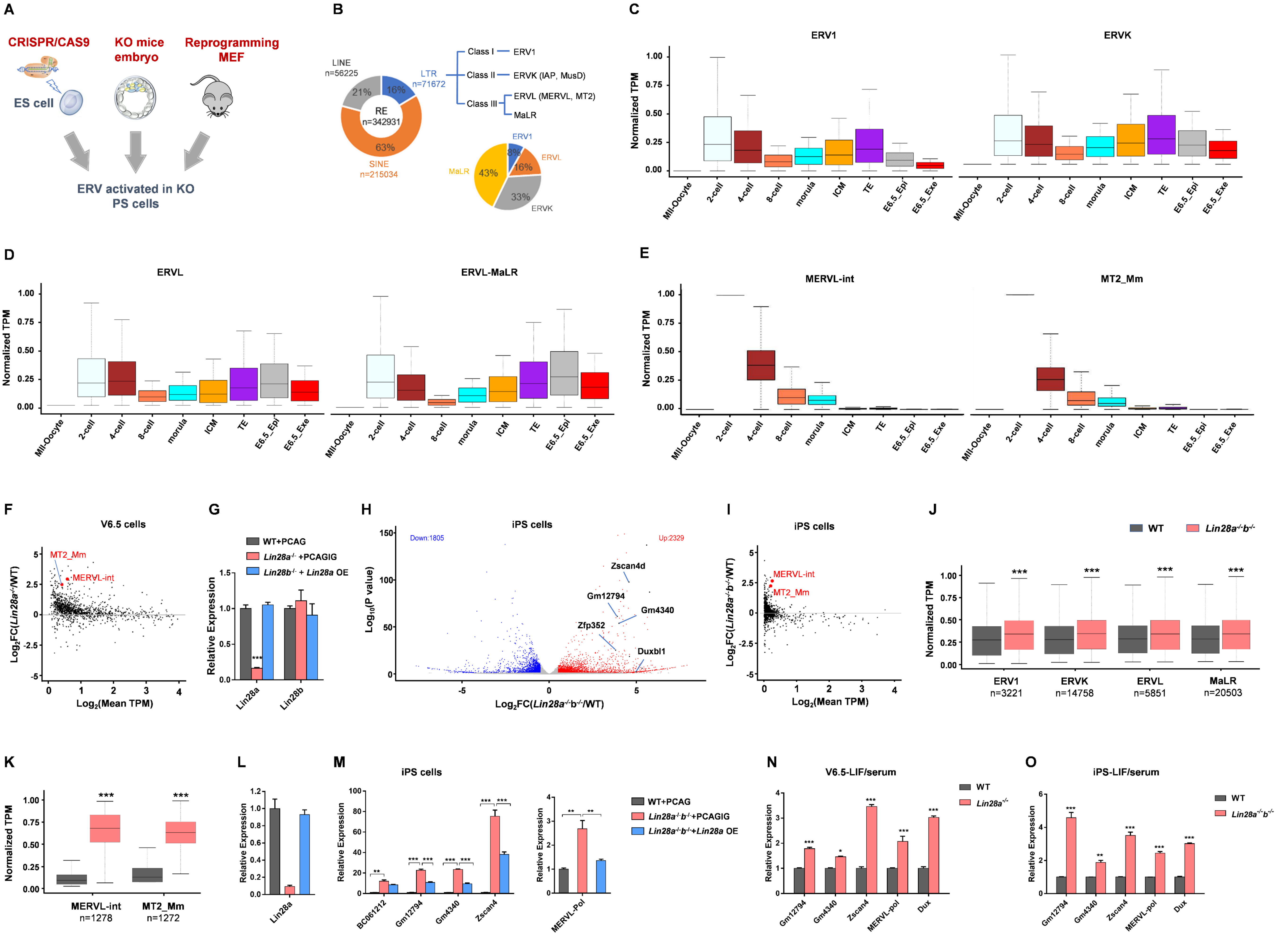
A
CRISPR/CAS9
KO mice embryo
Reprogramming MEF
ES cell
ERV activated in KO PS cells
B
LINE n=56225
LTR n=71672
RE n=342931
SINE n=215034
Class I — ERV1
Class II — ERVK (IAP, MusD)
Class III — ERVL (MERVL, MT2)
MaLR
ERV1
ERVL
MaLR
ERVK
C
ERV1
ERVK
Normalized TPM
MII-Oocyte
2-cell
4-cell
8-cell
morula
ICM
TE
E6.5_Epi
E6.5_Exe
D
ERVL
ERVL-MaLR
E
MERVL-int
MT2_Mm
F
V6.5 cells
MT2_Mm
MERVL-int
Log2FC(Lin28a-/-/WT)
Log2(Mean TPM)
G
WT+PCAG
Lin28a-/- +PCAGIG
Lin28b-/- + Lin28a OE
Relative Expression
Lin28a
Lin28b
H
iPS cells
Down:1805
Up:2329
Zscan4d
Gm12794
Gm4340
Zfp352
Duxbl1
Log10(P value)
Log2FC(Lin28a-/-b-/-/WT)
I
iPS cells
MERVL-int
MT2_Mm
J
WT
Lin28a-/-b-/-
ERV1 n=3221
ERVK n=14758
ERVL n=5851
MaLR n=20503
K
MERVL-int n=1278
MT2_Mm n=1272
L
M
iPS cells
BC061212
Gm12794
Gm4340
Zscan4
MERVL-Pol
WT+PCAG
Lin28a-/-b-/-+PCAGIG
Lin28a-/-b-/-+Lin28a OE
N
V6.5-LIF/serum
WT
Lin28a-/-
Dux
O
iPS-LIF/serum
Lin28a-/-b-/-

Fig. S2. LIN28 double knockout causes activation of ERV and 2C marker genes in iPS cells. Related to Fig. 2.

(A) A summary of three independent methods used to generate LIN28-deficient PS cells.

(B) Classification of transposable elements.

(C, D) Expression of different classes of ERV across different embryo developmental stages. The TPM values of LTR expression were linearly normalized to a range from 0 to 1 across all samples.

(E) Expression of two MERVL sub-classes MERVL-int and MT2_Mm in different embryo developmental stages. The TPM values of LTR expression were linearly normalized to a range from 0 to 1 across all samples.

(F) MA plot showing log2-fold changes in repeat sequence gene expression following Lin28a knockout in ES cells. Upregulated MERVL repeats are indicated.

(G) qRT-PCR showing Lin28a/b expression in the indicated conditions in ES cells.

(H) Volcano plot of RNA-seq showing alterations of gene expression upon Lin28a/b double knockout in iPS cells. Typical 2C genes are highlighted.

(I) MA plot showing log2-fold changes in repeat sequence gene expression following Lin28a/b double knockout in iPS cells.

(J) Different classes of Long Terminal Repeat or LTR expression in Lin28a/b double knockout iPS cells. The TPM values of LTR expression were linearly normalized to a range from 0 to 1 across all samples. ***: $p<0.001$, Wilcox signed rank test.

(K) Two sub-classes of MERVL genes MERVL-int and MT2_Mm expression in Lin28a/b double knockout iPS cells. The TPM values of LTR expression were linearly normalized to a range from 0 to 1 across all samples. ***: $p<0.001$, Wilcox signed rank test.

(L, M) qRT-PCR showing Lin28a (L), 2C genes and MERVL expression (M) in the indicated conditions in iPS cells.

(N, O) qRT-PCR showing the expression of 2C-related genes in wild-type and Lin28a knockout ES cells (N) and Lin28a/b double knockout iPS cells (O) cultured in LIF/serum medium for 7 days. For (N-P), *: $p<0.05$, **: $p<0.01$, ***: $p<0.001$, two-way ANOVA, $n=3$, error bar: standard error of the mean.

Figure S3

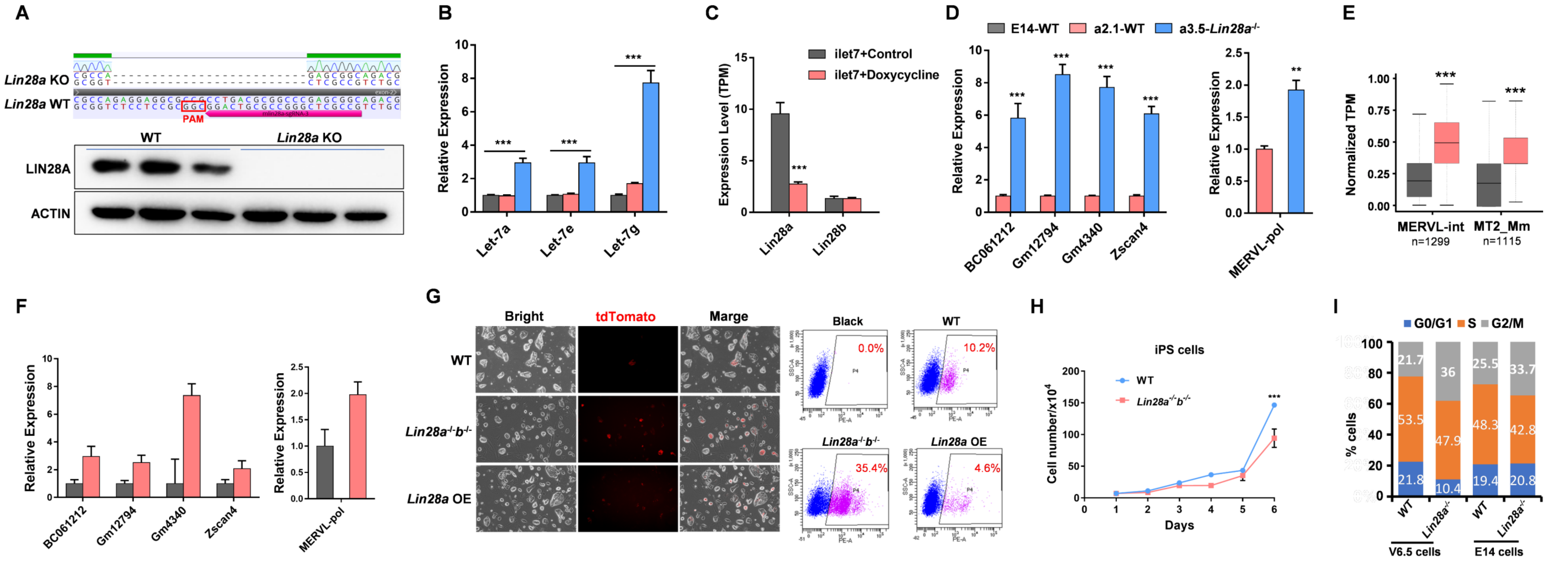

A
Lin28a KO
Lin28a WT
PAM
WT
Lin28a KO
LIN28A
ACTIN
B
Relative Expression
Let-7a
Let-7e
Let-7g
C
ilet7+Control
ilet7+Doxycycline
Expression Level (TPM)
Lin28a
Lin28b
D
E14-WT
a2.1-WT
a3.5-Lin28a-/-
Relative Expression
BC061212
Gm12794
Gm4340
Zscan4
MERVL-pol
E
Normalized TPM
MERVL-int
n=1299
MT2_Mm
n=1115
F
Relative Expression
BC061212
Gm12794
Gm4340
Zscan4
MERVL-pol
G
Bright
tdTomato
Marge
WT
Lin28a-/-b-/-
Lin28a OE
Black
0.0%
WT
10.2%
Lin28a-/-b-/-
35.4%
Lin28a OE
4.6%
H
iPS cells
WT
Lin28a-/-b-/-
Cell number/x10^4
Days
I
G0/G1
S
G2/M
% cells
21.7
36
25.5
33.7
53.5
47.9
48.3
42.8
21.8
10.4
19.4
20.8
WT
Lin28a-/-
V6.5 cells
E14 cells

Fig. S3. The increase of 2C marker genes and decrease of cell proliferation in *Lin28* deficiency cells. Related to Fig. 2.

(A) Sanger sequencing analysis and western blotting showing LIN28A protein knockout with CRISPR/CAS9 from multiple clones.

(B) qRT-PCR showing let-7 expression in un-transfected E14 ES cells, transfected wild-type clone a2.1 and CRISPR/CAS9 knockout clone a3.5.

(C) 2C gene and ERV expression in the same conditions as in (B).

(D) qRT-PCR showing Lin28 mRNA level in untreated and 3-day doxycycline-treated inducible let-7 (ilet7) ES cells. *: $p<0.05$, **: $p<0.01$, ***: $p<0.001$, two-way ANOVA, n=3, error bar: standard error of the mean.

(E) Analysis of RNA-seq showing two classes of MERVL genes MERVL-int and MT2_Mm are up-regulated in let-7 overexpressing ES cells. The TPM values of LTR expression were linearly normalized to a range from 0 to 1 across all samples. ***: $p<0.001$, Wilcox signed rank test.

(F) qRT-PCR showing 2C genes and ERV genes in let-7 overexpressing ES cells.

(G) Microscopy picture and flow cytometry data showing proportion of 2C-like cells using iPS cells transduced with a 2C::tdTomato reporter construct.

(H) Cell proliferation curve for wild-type and Lin28a/b double knockout iPS cells. ***: $p<0.001$, two-way ANOVA, n=3, error bar: standard error of the mean.

(I) Summary of cell cycle analysis across various conditions of Lin28 knockout cells and wild-type cells using PI staining and flow cytometry.

Figure S4

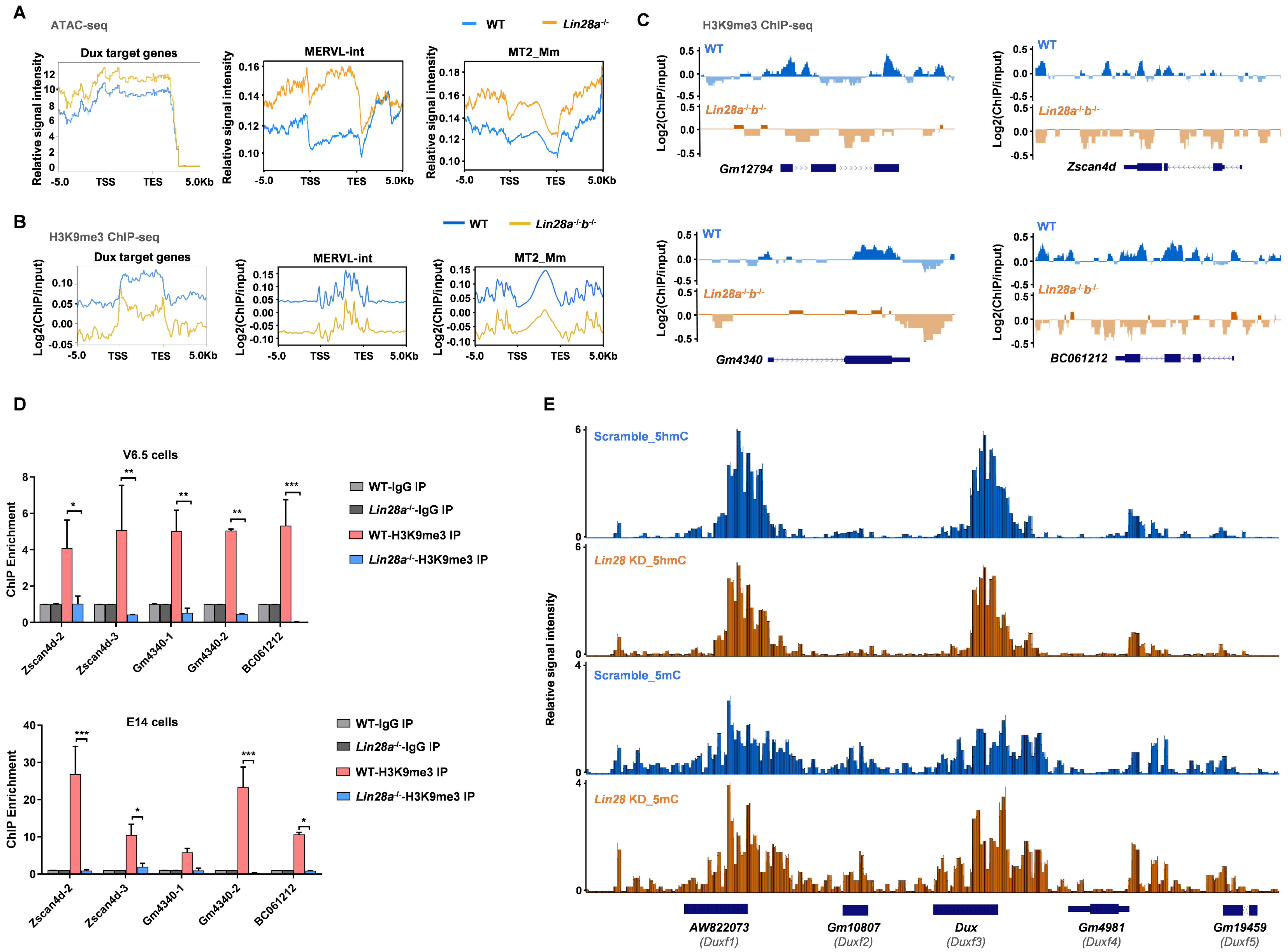

A
ATAC-seq
WT
Lin28a-/-
Dux target genes
MERVL-int
MT2_Mm
Relative signal intensity
TSS
TES
-5.0
5.0Kb
B
H3K9me3 ChIP-seq
WT
Lin28a-/-b-/-
Dux target genes
MERVL-int
MT2_Mm
Log2(ChIP/input)
C
H3K9me3 ChIP-seq
WT
Lin28a-/-b-/-
Gm12794
Zscan4d
Gm4340
BC061212
D
V6.5 cells
ChIP Enrichment
WT-IgG IP
Lin28a-/--IgG IP
WT-H3K9me3 IP
Lin28a-/--H3K9me3 IP
Zscan4d-2
Zscan4d-3
Gm4340-1
Gm4340-2
BC061212
E14 cells
E
Relative signal intensity
Scramble_5hmC
Lin28 KD_5hmC
Scramble_5mC
Lin28 KD_5mC
AW822073
(Duxf1)
Gm10807
(Duxf2)
Dux
(Duxf3)
Gm4981
(Duxf4)
Gm19459
(Duxf5)

Fig. S4. The epigenetic modification of Dux targets. Related to Fig. 3.

(A) ATAC-seq peaks in the proximity of the Dux target genes, MERVL-int and MT2_Mm ERVs in wild-type and knockout ES cells.

(B) ChIP-seq showing local H3K9me3 level in the proximity of Dux target genes, MERVL-int and MT2_Mm classes of ERVL genes in wild-type and double knockout iPS cells.

(C) UCSC Genome Browser view of ChIP-seq results for representative Dux targets 2C genes in wild-type and double knockout iPS cells.

(D) ChIP-PCR showing H3K9me3 levels of 2C genes in wild-type and CRISPR/CAS9 generated knockout V6.5 (left panel) or E14 ES cells (right panel).

(E) MeDIP-seq and 5hmC-seq showing levels of DNA methylation and hydroxymethylation in the proximity of Dux family genes in wild-type and Lin28a knockout ES cells. *: $p<0.05$, **: $p<0.01$, ***: $p<0.001$, two-way ANOVA, n=3, error bar: standard error of the mean.

Figure S5

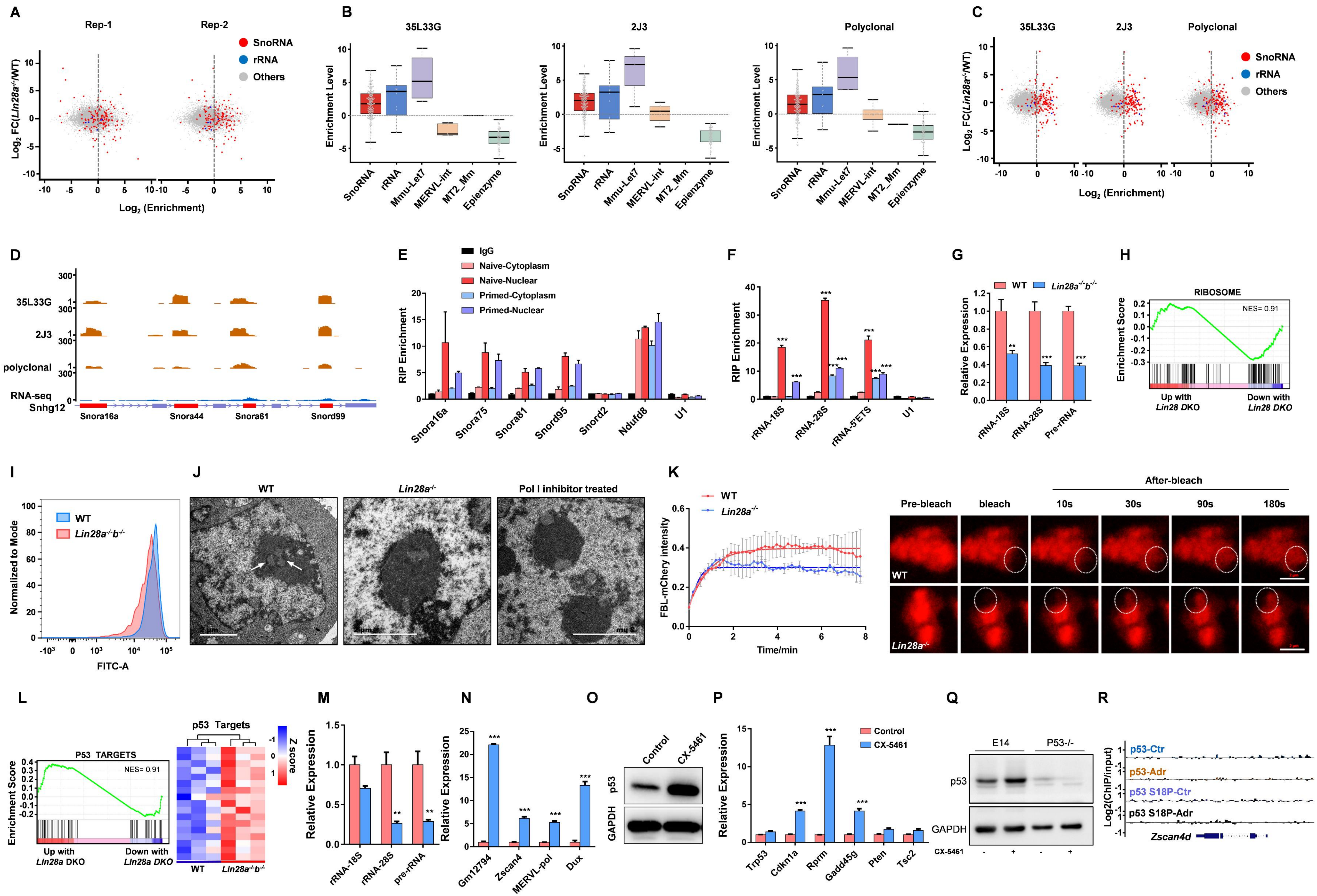

A
Rep-1
Rep-2
SnoRNA
rRNA
Others
Log2 FC(Lin28a-/-/WT)
Log2 (Enrichment)
B
35L33G
2J3
Polyclonal
Enrichment Level
SnoRNA
rRNA
Mmu-Let7
MERVL-int
MT2_Mm
Epienzyme
C
35L33G
2J3
Polyclonal
SnoRNA
rRNA
Others
Log2 FC(Lin28a-/-/WT)
Log2 (Enrichment)
D
35L33G
2J3
polyclonal
RNA-seq
Snhg12
Snora16a
Snora44
Snora61
Snord99
E
IgG
Naive-Cytoplasm
Naive-Nuclear
Primed-Cytoplasm
Primed-Nuclear
RIP Enrichment
Snora16a
Snora75
Snora81
Snord95
Snord2
Ndufd8
U1
F
RIP Enrichment
rRNA-18S
rRNA-28S
rRNA-5'ETS
U1
G
WT
Lin28a-/-b-/-
Relative Expression
rRNA-18S
rRNA-28S
Pre-rRNA
H
RIBOSOME
NES= 0.91
Enrichment Score
Up with Lin28 DKO
Down with Lin28 DKO
I
WT
Lin28a-/-b-/-
Normalized to Mode
FITC-A
J
WT
Lin28a-/-
Pol I inhibitor treated
K
WT
Lin28a-/-
FBL-mCherry intensity
Time/min
After-bleach
Pre-bleach
bleach
10s
30s
90s
180s
WT
Lin28a-/-
L
P53 TARGETS
NES= 0.91
Enrichment Score
Up with Lin28a DKO
Down with Lin28a DKO
p53 Targets
Z score
WT
Lin28a-/-b-/-
M
Relative Expression
rRNA-18S
rRNA-28S
pre-rRNA
N
Relative Expression
Gm12794
Zscan4
MERVL-pol
Dux
O
Control
CX-5461
p53
GAPDH
P
Control
CX-5461
Relative Expression
Trp53
Cdkn1a
Rprm
Gadd45g
Pten
Tsc2
Q
E14
P53-/-
p53
GAPDH
CX-5461
R
p53-Ctr
p53-Adr
p53 S18P-Ctr
p53 S18P-Adr
Log2(ChIP/input)
Zscan4d

Fig. S5. Lin28 deficiency leads to nucleolar abnormalities. Related to Fig. 4.

(A) Enrichment of LIN28A binding targets and fold change expression between Lin28a knockdown and scramble are plotted for snoRNA, rRNA and all other genes.

(B) Analysis of published CLIP-seq data (Cho et. al., 2012) with three different LIN28A antibodies (35L33G, 2J3 and Polyclonal antibodies) showing enrichment of binding to indicated RNA. Let7 was used as positive control.

(C) Enrichment of LIN28A binding targets and change expression between Lin28a knockdown and scramble (Cho et. al., 2012) are plotted for snoRNA, rRNA and all other genes.

(D) An example of LIN28 binding signal derived from CLIP-seq at the indicated snoRNA locus.

(E) RIP-qPCR showing LIN28 binding to snoRNAs, as well as its cytoplasmic binding target Ndufd8 in the naïve and primed ES cells.

(F) RIP-qPCR analysis of LIN28A binding to rRNA in the naïve and primed ES cells.

(G) qRT-PCR showing the expression of pre- and mature rRNA in wild-type and Lin28a/b double knockout iPS cells.

(H) GSEA analysis showing collective changes in the ribosomal protein gene set of Lin28a/b double knockout iPS cells versus wild-type cells.

(I) Flow cytometry showing OP-puromycin labeling of wild-type and Lin28a/b double knockout iPS cells.

(J) Electro-microscopy showing nuclear morphology of wild-type and Lin28a knockout E14 ES cells.

(K) Left panel: Normalized fluorescence intensity in wild-type and Lin28 knockout cells stably expressing FBL-mCherry with FRAP analysis. Right panel, representative time-lapse images of pre-bleaching, immediately after bleaching (Bleach) and the first 180 s after bleaching were shown. Curves are given as mean values $\pm$ SD; n = 4 biological repeats representing at least 9 different cells.

(L) Left panel: GSEA analysis showing collective changes in the p53 pathway gene set of Lin28a/b double knockout iPS cells versus wild-type cells. Right panel: up-regulation of p53 pathway genes in Lin28a/b double knockout iPS cells compared with wild-type cells.

(M) qRT-PCR showing the expression of 18S, 28S and pre-rRNA in ES cells treated with 4 μM Pol I inhibitor (CX-5461) for 12 hr or equal water (control).

(N) qRT-PCR showing 2C gene expression in ES cells treated with 4 μM Pol I inhibitor (CX-5461) for 12 hr or equal water (control).

(O) Western blotting showing activation of p53 with treatment of CX-5461 or Nutin3 as a control.

(P) qPCR showing p53 target expression upon CX-5461 treatment.

(Q) Western blotting showing p53 protein level upon treatment in wild-type and p53 knockout cells.

(R) UCSC Genome Browser view of ChIP-seq results using antibodies of p53 and phosphorylated p53 S18P for Zscan4d gene locus. *: $p<0.05$, **: $p<0.01$, ***: $p<0.001$, two-way ANOVA, n=3, error bar: standard error of the mean.

Figure S6

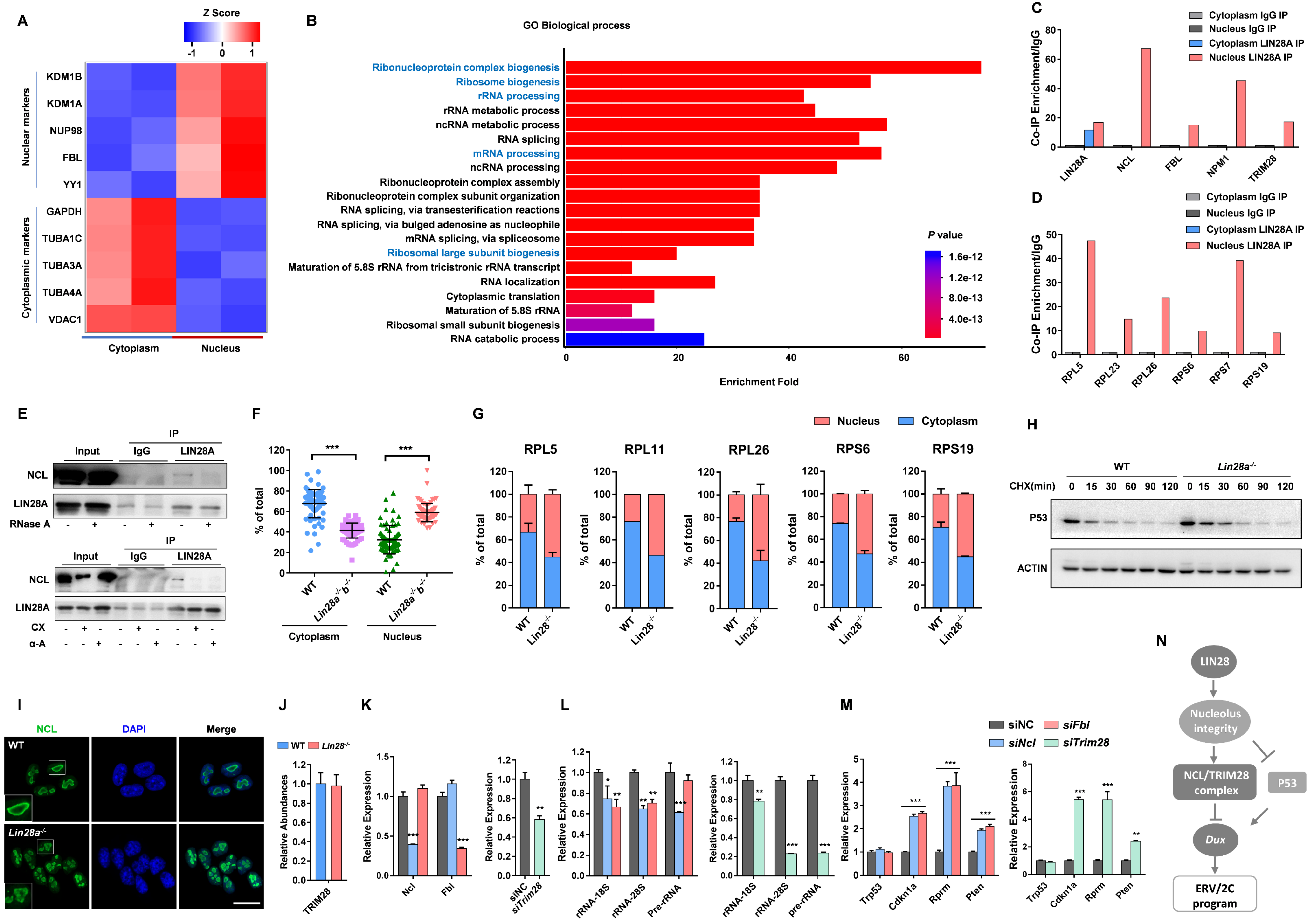
A
Z Score
Nuclear markers
KDM1B
KDM1A
NUP98
FBL
YY1
Cytoplasmic markers
GAPDH
TUBA1C
TUBA3A
TUBA4A
VDAC1
Cytoplasm
Nucleus
B
GO Biological process
Ribonucleoprotein complex biogenesis
Ribosome biogenesis
rRNA processing
rRNA metabolic process
ncRNA metabolic process
RNA splicing
mRNA processing
ncRNA processing
Ribonucleoprotein complex assembly
Ribonucleoprotein complex subunit organization
RNA splicing, via transesterification reactions
RNA splicing, via bulged adenosine as nucleophile
mRNA splicing, via spliceosome
Ribosomal large subunit biogenesis
Maturation of 5.8S rRNA from tricistronic rRNA transcript
RNA localization
Cytoplasmic translation
Maturation of 5.8S rRNA
Ribosomal small subunit biogenesis
RNA catabolic process
P value
1.6e-12
1.2e-12
8.0e-13
4.0e-13
Enrichment Fold
C
Co-IP Enrichment/IgG
Cytoplasm IgG IP
Nucleus IgG IP
Cytoplasm LIN28A IP
Nucleus LIN28A IP
LIN28A
NCL
FBL
NPM1
TRIM28
D
RPL5
RPL23
RPL26
RPS6
RPS7
RPS19
E
IP
Input
IgG
LIN28A
NCL
RNase A
CX
α-A
F
% of total
WT
Lin28a-/-b-/-
Cytoplasm
Nucleus
G
Nucleus
Cytoplasm
RPL5
RPL11
RPL26
RPS6
RPS19
Lin28-/-
H
WT
Lin28a-/-
CHX(min)
P53
ACTIN
I
NCL
DAPI
Merge
J
Lin28-/-
Relative Abundances
TRIM28
K
Relative Expression
Ncl
Fbl
siNC
siTrim28
L
rRNA-18S
rRNA-28S
Pre-rRNA
pre-rRNA
M
siNC
siFbl
siNcl
siTrim28
Trp53
Cdkn1a
Rprm
Pten
N
LIN28
Nucleolus integrity
NCL/TRIM28 complex
P53
Dux
ERV/2C program

Fig. S6. *Lin28* interacts with nucleolar proteins and regulates *Dux* expression through the NCL/TRIM28 complex. Related to Fig. 5.

(A) Heatmap showing that cytosol and nucleus fractions were separated for proteomics analysis.

(B) GO analysis of proteins pulled down by the LIN28A antibody.

(C, D) Co-immunoprecipitation (Co-IP) showing the interaction between endogenous LIN28A and nucleolus proteins (C) and ribosomal proteins (D) in ES cells by mass spectroscopy.

(E) Co-IP showing association of endogenous LIN28A and NCL proteins in ES cells treated with RNase A (100 μg/ml for 30 min at 37 ℃, upper panel) or Pol I inhibitor (4 μM CX-5461 for 12 hr), Pol II inhibitor (10 μg/ml α-Amantin for 12 hr) (lower panel) by Western blotting.

(F, G) Proteomics analysis showing the nucleus and cytoplasm proportion of all ribosomal proteins (F) and indicated proteins (G) in wild-type and Lin28 knockout cells. (G) is the average of Lin28a knockout ES cells and Lin28a/b knockout iPS cells. ***: $p<0.001$, t-test, error bar: standard error of the mean. Error bar in (G) means standard error of the mean of 2 independent proteomics experiments from ES and iPS cells.

(H) Western blot showing p53 protein half-life in wild-type and Lin28a knockout ES cells treated with 100 μg/ml of cycloheximide (CHX).

(I) Immunofluorescence staining of NCL in wild-type and Lin28a/b double knockout iPS cells. Scale bar, 20 μm.

(J) Proteomics analysis showing the relative abundances of TRIM28 proteins in wild-type and Lin28 knockout cells (averaged proteomics data from Lin28a knockout ES cells and Lin28a/b knockout iPS cells). Error bar: standard error of the mean. n=2 independent proteomics experiments.

(K-M) qRT-PCR showing the mRNA levels of indicated genes in ES cells transfected with scramble negative control or siRNAs against Ncl, Fb or Trim28. *: $p<0.05$, **: $p<0.01$, ***: $p<0.001$, two-way ANOVA, n=3, error bar: standard error of the mean.

(N) Schematic illustration showing LIN28 regulates Dux expression through the nucleolus and NCL/TRIM28 complex.

Figure S7

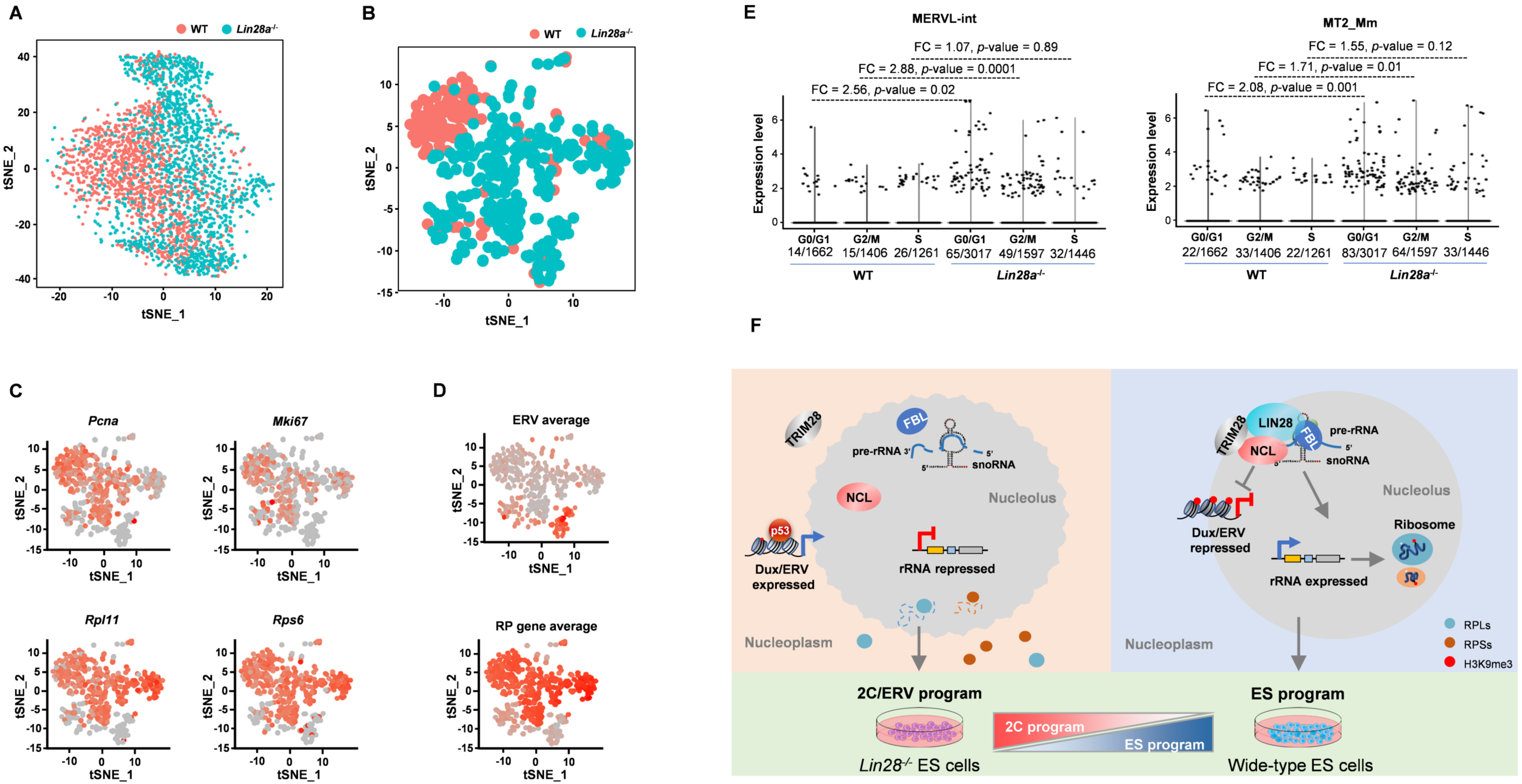
A
WT
Lin28a-/-
tSNE_1
tSNE_2
B
C
Pcna
Mki67
Rpl11
Rps6
D
ERV average
RP gene average
E
MERVL-int
MT2_Mm
FC = 1.07, p-value = 0.89
FC = 2.88, p-value = 0.0001
FC = 2.56, p-value = 0.02
FC = 1.55, p-value = 0.12
FC = 1.71, p-value = 0.01
FC = 2.08, p-value = 0.001
Expression level
G0/G1 14/1662
G2/M 15/1406
S 26/1261
G0/G1 65/3017
G2/M 49/1597
S 32/1446
G0/G1 22/1662
G2/M 33/1406
S 22/1261
G0/G1 83/3017
G2/M 64/1597
S 33/1446
F
TRIM28
FBL
pre-rRNA
snoRNA
NCL
Nucleolus
p53
Dux/ERV expressed
rRNA repressed
Nucleoplasm
LIN28
Dux/ERV repressed
Ribosome
rRNA expressed
RPLs
RPSs
H3K9me3
2C/ERV program
ES program
2C program
Lin28-/- ES cells
Wide-type ES cells

Fig. S7. Single cell RNA-seq analysis of wild-type and *Lin28* knockout ES cells. Related to Fig. 6.

(A) Single cell RNA-seq analysis and tSNE plot showing clustering of wild-type and Lin28 knockout ES cells.

(B) tSNE plot showing clustering of wild-type and Lin28 knockout ES cells filtered to only leave those with detected ERV expression.

(C) tSNE plots showing indicated gene expression with cells filtered to only leave those with detected ERV expression.

(D) tSNE plots showing averaged ERV or RP gene expression with cells filtered to only leave those with detected ERV expression.

(E) Single cell RNA-seq analysis showing cell cycle phase distribution of those cells expressing MERVL-int or MT2_Mm in wild-type and Lin28a knockout ES cells. Each dot represents a single cell. The numbers below reflect the percentage of cells with ERV detected in each cell cycle phase. FC: fold changes of the number of cells in a certain cell cycle phase between knockout and wild-type cells. Fold changes for G0/G1 and G2/M phases are significant, but not for S phase. The p-values were calculated by Fisher's exact test.

(F) Schematic illustration showing LIN28 regulates nucleolar functions to coordinate 2C and ES cell programs. In the wild-type ES cell program, LIN28 in the nucleolus resides in the same complex with NCL/TRIM28 or in the liquid phase with other nucleolar RNAs and RNA binding proteins, and mediates NCL/TRIM28 occupancy on the Dux locus to repress Dux expression. Meanwhile, LIN28 also facilitates the same complex/liquid phase to promote rRNA expression and ribosome biogenesis. In contrast, in the Lin28 knockout ES cells, as the LIN28-mediated complex is interrupted, Dux repression is released and rRNA expression is reduced which both contribute to the 2-cell like cell program. Nucleolar stress also activates p53 which can directly bind to Dux locus and promotes Dux expression.
